# Injectable Biopolymer-Based Hydrogels: A Next-Generation Platform for Minimally Invasive Therapeutics

**DOI:** 10.3390/gels11060383

**Published:** 2025-05-23

**Authors:** Nargish Parvin, Sang Woo Joo, Tapas Kumar Mandal

**Affiliations:** 1 School of Mechanical Engineering, Yeungnam University, Gyeongsan 38541, Republic of Korea; nargish.parvin@gmail.com; 2 School of Chemical Engineering, Yeungnam University, Gyeongsan 38541, Republic of Korea

**Keywords:** injectable hydrogels, biopolymer-based gels, targeted drug delivery, tissue regeneration, in situ gelation

## Abstract

Injectable biopolymer-based hydrogels have emerged as a powerful class of biomaterials designed for minimally invasive therapeutic strategies in modern medicine. These smart hydrogels, derived from natural biopolymers, such as alginate, chitosan, gelatin, hyaluronic acid, and collagen, offer unique advantages, including biocompatibility, biodegradability, and the ability to mimic the extracellular matrix. This review provides a comprehensive overview of recent advancements in the design, crosslinking mechanisms, and biofunctionality of injectable hydrogels tailored for targeted drug delivery and tissue regeneration. Special attention is given to their role in in situ gelling systems, cancer therapy, musculoskeletal repair, and neural regeneration. Challenges related to mechanical strength, degradation control, and clinical translation are also discussed, along with future perspectives for scalable manufacturing and regulatory approval.

## 1. Introduction

### 1.1. Background and Significance of Injectable Hydrogels

The concept of minimally invasive therapeutics has revolutionized modern clinical practice, driving the need for smart materials capable of adapting to dynamic physiological conditions while maintaining therapeutic efficacy. Injectable hydrogels have emerged as one such advanced class of biomaterials, offering remarkable utility in this regard. These soft, water-swollen, three-dimensional polymeric networks can be administered in a liquid state and undergo sol-to-gel transition under physiological conditions, enabling in situ formation at the target site. This not only minimizes surgical invasiveness but enhances localized therapeutic delivery, reduces systemic side effects, and improves patient compliance [1]. Historically, the development of hydrogels can be traced back to the 1960s when poly(2-hydroxyethyl methacrylate) hydrogels were first introduced for biomedical use [2]. Since then, the hydrogel field has undergone exponential growth, progressing from inert polymer matrices to highly tunable, bioresponsive, and multifunctional platforms capable of mimicking native tissue environments. Injectable hydrogels represent a significant evolution in this trajectory due to their ability to conform to complex geometries, support cellular infiltration, and provide mechanical support during tissue healing and regeneration. The relevance of injectable hydrogels is particularly pronounced in applications, such as drug delivery, tissue engineering, wound healing, regenerative medicine, and even immunotherapy. Their unique physicochemical properties, such as high water content, adjustable viscoelasticity, and tunable degradation kinetics, make them highly versatile platforms for encapsulating and releasing therapeutic agents in a sustained and localized manner [3,4]. Additionally, these hydrogels can be engineered to respond to specific biological stimuli, including pH, temperature, or enzymatic activity, making them ideal for controlled and on-demand drug release [5,6]. A critical factor underpinning the increasing clinical interest in injectable hydrogels is their potential to replicate the mechanical and biochemical features of the extracellular matrix (ECM), thus providing a favorable microenvironment for cell growth and tissue development? In regenerative applications, this ECM-mimicking feature becomes indispensable for cell migration, adhesion, differentiation, and proliferation [7]. Furthermore, the ability to incorporate bioactive molecules, growth factors, and living cells into hydrogel matrices has broadened their applicability in cell therapy and tissue scaffolding, aligning with the goals of personalized and precision medicine. Importantly, injectable hydrogels provide a modular platform that can be tailored for specific biomedical needs through variations in polymer composition, crosslinking strategy (physical or chemical), and incorporation of functional agents. These design attributes allow for broad adaptability in clinical settings, especially in difficult-to-access tissues or organs, such as the central nervous system, cartilage, or cardiovascular system [8]. With the global burden of chronic diseases and degenerative conditions on the rise, the demand for non-invasive therapeutic platforms continues to grow. Injectable hydrogels are at the forefront of this demand, offering a pathway to site-specific treatment, enhanced therapeutic retention, and reduced recovery times. The surge in publications, patents, and commercial products over the past decade attests to their growing clinical importance and translational potential [9]. Despite their promise, challenges persist regarding the mechanical robustness of hydrogels, rapid degradation in vivo, and issues related to sterilization, storage, and large-scale manufacturing. Nevertheless, continuous advancements in polymer chemistry, materials science, and biomedical engineering are progressively addressing these issues, making injectable hydrogels a cornerstone in the next generation of therapeutic technologies.

### 1.2. Motivation for Using Biopolymer-Based Gels in Medicine

The motivation behind using biopolymer-based injectable hydrogels in medicine lies in the intersection of biocompatibility, biodegradability, and biomimicry features that are crucial for safe and effective biomedical interventions. Unlike synthetic hydrogels, which may induce inflammatory responses or require complex chemical modifications, natural biopolymers offer intrinsic advantages due to their biological origin and physiological compatibility [10]. Biopolymers, such as alginate, chitosan, hyaluronic acid (HA), gelatin, and collagen, have been extensively studied as the building blocks for hydrogels. These materials are either directly extracted from natural sources or derived through simple modifications of naturally occurring macromolecules, ensuring minimal cytotoxicity and immunogenicity [11]. For example, chitosan, derived from the deacetylation of chitin, possesses inherent antibacterial and wound-healing properties, making it suitable for dermal applications [12]. Similarly, HA, a component of the human ECM, promotes cell proliferation and migration, which are vital for tissue regeneration [13]. Another compelling motivation is the enzymatic or hydrolytic degradability of biopolymer-based hydrogels, allowing for predictable degradation and resorption post-therapy. This eliminates the need for surgical removal, especially critical in pediatric or geriatric populations where secondary surgeries pose increased risks. Moreover, the degradation products of these biopolymers are typically non-toxic and easily metabolized by the body, aligning with the principles of biosafety and sustainability [14]. Functionally, biopolymer-based hydrogels protablevide a conducive milieu for therapeutic payload delivery. Their physicochemical nature allows for the encapsulation of a wide range of therapeutic agents, including small molecules, proteins, peptides, and nucleic acids. By modulating network density, porosity, and crosslinking mechanisms, controlled release profiles can be achieved, reducing the frequency of administration and improving therapeutic outcomes [15]. For instance, gelatin-based hydrogels can be engineered to exhibit temperature-sensitive sol–gel transitions, facilitating minimally invasive injection and subsequent gelation at body temperature [16]. In the realm of regenerative medicine, biopolymers offer a matrix that supports cellular functions, largely because their biochemical cues resemble the native ECM. This attribute is crucial for applications, like cartilage and bone repair, where scaffolds must not only support mechanical load but stimulate osteo- or chondrogenesis [17]. Moreover, biopolymers are highly amenable to functionalization with peptides, growth factors, and nanoparticles, thus enhancing their therapeutic efficacy and allowing for multi-modal treatments within a single platform [18] see in Table 1.

The surge in antimicrobial resistance, cancer recurrence, and neurodegenerative disorders has also steered research towards biopolymer-based hydrogels for localized, sustained, and multi-functional therapies. In oncology, injectable hydrogels loaded with chemotherapeutics or immune modulators can be used to create a depot at the tumor site, reducing systemic toxicity while enhancing drug concentration locally [19]. Likewise, in neural applications, soft hydrogels derived from biopolymers minimize mechanical mismatch with delicate brain tissues, mitigating inflammatory responses and promoting regeneration [20]. Lastly, environmental and regulatory considerations further motivate the adoption of biopolymer-based systems. Derived from renewable resources, these materials contribute to the sustainability of biomedical products. Their well-established safety profiles facilitate faster regulatory approval pathways compared to synthetic alternatives, thereby accelerating clinical translation [21].

### 1.3. Objectives and Scope of the Review

The overarching objective of this review is to critically examine the current landscape of injectable biopolymer-based hydrogels, focusing on their design principles, functional mechanisms, and biomedical applications. While injectable hydrogels, in general, have been widely discussed in the literature, there exists a compelling need to specifically address those derived from natural biopolymers due to their superior biofunctionality, regulatory advantages, and emerging clinical relevance. This review aims to provide a comprehensive yet focused synthesis of the most recent and impactful advancements in the field. Particular emphasis is placed on the following:

- The physicochemical properties of natural biopolymers and their transformation into injectable hydrogel systems.
- Various crosslinking mechanisms—both physical (e.g., ionic, thermal, or hydrogen bonding) and chemical (e.g., enzymatic or photo-crosslinking) that facilitate in situ gelation under physiological conditions.
- Biofunctional characteristics of these hydrogels, including responsiveness to environmental stimuli, drug-loading capacity, degradation kinetics, and support for cellular activities.
- Biomedical applications spanning targeted drug delivery, tissue engineering, neural regeneration, cancer therapy, and musculoskeletal repair.

In addition to highlighting the therapeutic potential, this review critically evaluates the challenges associated with clinical translation, such as insufficient mechanical stability, immunogenicity, sterilization difficulties, and scalability. Strategies to address these limitations through composite formulations, hybrid crosslinking strategies, and nanomaterial integration are also explored. The scope extends to future directions in the field, touching upon emerging technologies, such as 3D bioprinting, microneedle-based delivery systems, and smart hydrogels integrated with biosensors for theranostic applications. This review also reflects on regulatory frameworks and commercialization strategies, which are essential for moving laboratory innovations to clinical practice. By consolidating multidisciplinary insights from materials science, bioengineering, and clinical research, this review intends to serve as a valuable resource for researchers, clinicians, and industry professionals aiming to develop next-generation injectable hydrogel platforms tailored for minimally invasive therapeutics.

**Figure 1:**
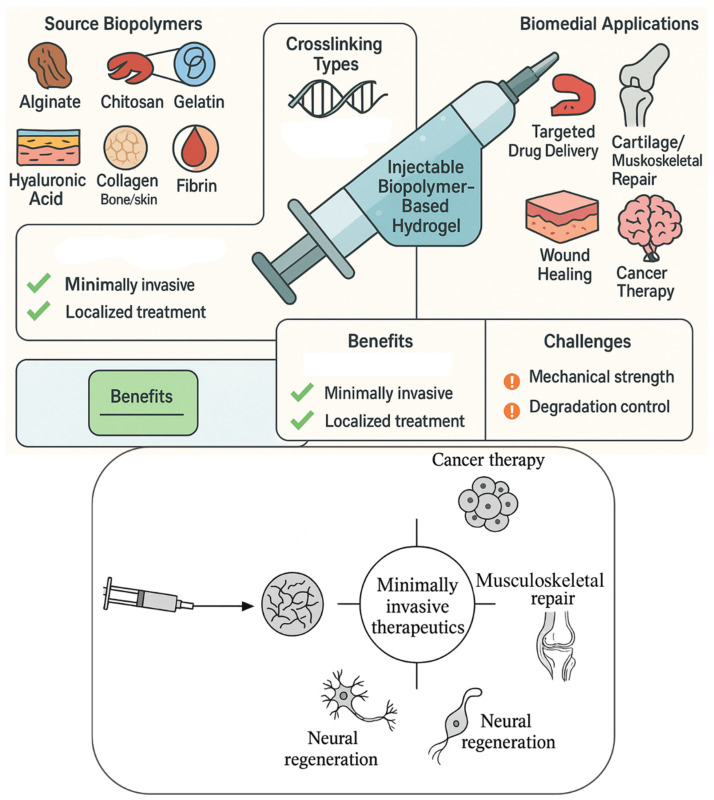
Schematic Illustration of Injectable Biopolymer-Based Hydrogels for Minimally Invasive Therapeutics.

Figure 1 illustrates the composition, crosslinking mechanisms, and biomedical applications of injectable biopolymer-based hydrogels. Natural biopolymers, such as alginate, chitosan, gelatin, hyaluronic acid, collagen, and silk fibroin, are derived from biological sources and serve as bioactive building blocks. These polymers undergo physical (e.g., thermal, ionic) or chemical (e.g., photo, enzymatic) crosslinking to form in situ hydrogels. Upon injection into target sites, these hydrogels conform to irregular tissue geometry, offering controlled drug release, enhanced cellular infiltration, and tissue regeneration. The applications span cancer therapy, neural repair, musculoskeletal regeneration, wound healing, and localized drug delivery. The illustration highlights the biocompatibility, biodegradability, and ECM-mimicking potential of biopolymer hydrogels in minimally invasive biomedical interventions.

## 2. Classification of Biopolymers for Injectable Hydrogels

Injectable hydrogels are primarily constructed from biopolymers, which can be broadly categorized into natural and synthetic types. Natural biopolymers, derived from biological sources, are particularly attractive due to their inherent biocompatibility, biodegradability, and ability to mimic the extracellular matrix (ECM). This section focuses on the most prominent natural biopolymers used in injectable hydrogel systems.

### 2.1. Natural Biopolymers

Natural biopolymers serve as excellent candidates for hydrogel fabrication due to their structural resemblance to biological tissues. They often contain functional groups conducive to chemical or physical crosslinking, facilitating hydrogel formation under physiological conditions. Key natural polymers used in injectable hydrogels include alginate, chitosan, gelatin, hyaluronic acid, and collagen. Alginate is a naturally occurring anionic polysaccharide extracted primarily from brown seaweed. It consists of alternating blocks of α-L-guluronic acid (G) and β-D-mannuronic acid (M), with the G-blocks being primarily responsible for ionic crosslinking in the presence of divalent cations, like calcium (Ca^2^^+^) [22]. Alginate hydrogels form rapidly upon contact with these ions, making them highly suitable for in situ gelation in injectable applications. One of the major advantages of alginate-based hydrogels is their mild gelation process, which preserves the integrity of encapsulated cells and drugs. Their porous structure facilitates nutrient diffusion, making them favorable for tissue regeneration, especially in cartilage and bone repair [23]. However, alginate lacks inherent cell-adhesion motifs, often necessitating chemical modification or blending with bioactive molecules to improve cellular interactions [24]. Chitosan is a cationic linear polysaccharide derived from the deacetylation of chitin, a major component of crustacean shells. It is biocompatible, biodegradable, and exhibits inherent antimicrobial properties [25]. Its ability to form hydrogels is mainly governed by its solubility in acidic environments and its crosslinking with agents, such as genipin, β-glycerophosphate, or tripolyphosphate (TPP). Due to its mucoadhesive nature, chitosan is extensively used for mucosal drug delivery and wound healing. It also supports neural regeneration and hemostatic applications [26]. However, chitosan hydrogels may require pH adjustments or additional agents to gel effectively under physiological conditions, which can complicate clinical translation [27]. Gelatin is a partially hydrolyzed form of collagen and retains many of the bioactive sequences responsible for cellular recognition and adhesion, such as the RGD (Arg-Gly-Asp) motif [28]. It is thermoresponsive, forming a gel upon cooling, and is often chemically modified (e.g., GelMA: gelatin methacryloyl) to enable photo-crosslinking for enhanced stability. Gelatin-based injectable hydrogels have demonstrated promising results in soft tissue engineering, drug delivery, and cell encapsulation applications [29]. Its degradation can be finely tuned by enzymatic activity (e.g., matrix metalloproteinases), making it highly suitable for tissue remodeling environments. Nonetheless, gelatin hydrogels suffer from low mechanical strength and rapid degradation unless crosslinked with stabilizers [30]. Hyaluronic acid (HA) is a naturally occurring non-sulfated glycosaminoglycan composed of repeating disaccharide units of D-glucuronic acid and N-acetyl-D-glucosamine [31]. As a major component of the ECM, HA is involved in various cellular processes, including proliferation, migration, and wound repair. Injectable hydrogels based on HA often involve chemical modifications (e.g., thiolation, methacrylation) to allow for tunable crosslinking and improved mechanical properties [32]. HA-based hydrogels have been utilized in a range of applications, such as dermal fillers, osteoarthritis treatment, and as vehicles for localized drug and gene delivery [33]. However, the rapid enzymatic degradation of unmodified HA in vivo necessitates crosslinking strategies to prolong its therapeutic effect [34]. Collagen, particularly Type I, is the most abundant protein in the human body and plays a critical role in structural integrity and tissue regeneration. It can self-assemble into fibrillar networks under physiological conditions, allowing for injectable gel formation without harsh chemical crosslinkers [35]. Collagen provides native binding sites for cells and supports angiogenesis and matrix deposition in engineered tissues. It is widely used in wound dressings, bone grafts, and cardiac patches, owing to its excellent biocompatibility and biofunctionality [36]. However, concerns remain regarding its immunogenicity (depending on source), batch variability, and mechanical weakness in hydrated environments, often requiring composite strategies or crosslinking for clinical applications [37].

### 2.2. Semi-Synthetic and Hybrid Biopolymers

Semi-synthetic and hybrid biopolymers have emerged as a promising class of materials that bridge the gap between natural and synthetic systems. These materials integrate the biological advantages of natural polymers, such as cell recognition and enzymatic degradability, with the tailorable physical and chemical properties of synthetic counterparts, such as improved mechanical strength, tunable degradation profiles, and stimuli-responsive behavior [38]. This unique hybridization addresses many of the limitations faced by purely natural biopolymers in injectable hydrogel systems, including uncontrolled gelation kinetics, insufficient mechanical resilience, and batch-to-batch variability. Semi-synthetic hydrogels are generally derived by chemically modifying natural polymers or by blending natural and synthetic polymers. Common modification techniques include methacrylation, oxidation, amidation, and thiolation [39]. For example, gelatin methacryloyl (GelMA) is a semi-synthetic derivative of gelatin widely used for photopolymerizable hydrogel systems [40]. Similarly, hyaluronic acid can be functionalized with hydrazide or aldehyde groups to enable Schiff base or Michael addition reactions, allowing injectable systems to gel in situ under physiological conditions [41]. These chemical alterations permit precise control over gelation time, crosslinking density, swelling behavior, and degradation rates, all of which are critical for tailoring injectable hydrogels for specific therapeutic applications, such as cartilage repair, drug delivery, and 3D cell encapsulation [42]. Hybrid hydrogels, a combination of alginate (natural) and polyethylene glycol (PEG, synthetic), create a system with the ECM-mimicking properties of alginate and the mechanical robustness of PEG [43]. Such systems often allow dual-mode crosslinking, where one polymer forms a rapid ionic network, and the other contributes to long-term mechanical stability via covalent bonding. This hybrid design allows the hydrogels to demonstrate a multi-functional performance, including resistance to premature degradation, cytocompatibility, controlled drug release, and appropriate rheological properties for injection through narrow-gauge needles [44]. These features make them highly suitable for applications in minimally invasive orthopedic treatments, spinal cord injury repair, and localized cancer therapy [45]. An exciting advancement in semi-synthetic and hybrid systems is the incorporation of stimuli-responsive features. Functional groups or nanoparticles can be embedded within these matrices to respond to external triggers, such as pH, temperature, enzymes, light, or magnetic fields [46]. For example, thermoresponsive hybrid hydrogels combining chitosan and β-glycerophosphate remain in a liquid state at room temperature but rapidly solidify at body temperature, forming in situ gels post-injection [47]. Other smart systems involve photo-crosslinkable hybrids, such as GelMA/PEGDA (polyethylene glycol diacrylate), enabling rapid UV-induced gelation after injection, allowing for spatiotemporal control over gel stiffness and encapsulated therapeutic release [48]. Despite their promise, semi-synthetic and hybrid hydrogels also pose certain challenges. Residual chemical groups or photoinitiators can potentially trigger cytotoxic responses if not thoroughly removed. Moreover, the complex synthesis routes and quality control of these materials may hinder their scalability and regulatory approval for clinical translation [49]. Additionally, reproducibility in large-scale production and long-term biocompatibility still need comprehensive investigation. Numerous preclinical and early clinical studies are underway using semi-synthetic hydrogels for wound healing, ocular therapies, and tumor inhibition [50]. A notable example includes the use of hyaluronic acid–tyramine conjugates for injectable ophthalmic formulations, and GelMA-based hydrogels in cardiac regeneration scaffolds [51]. These developments suggest that, with proper optimization, semi-synthetic and hybrid hydrogels could significantly advance the field of personalized, injectable therapies in regenerative medicine.

**Figure 2:**
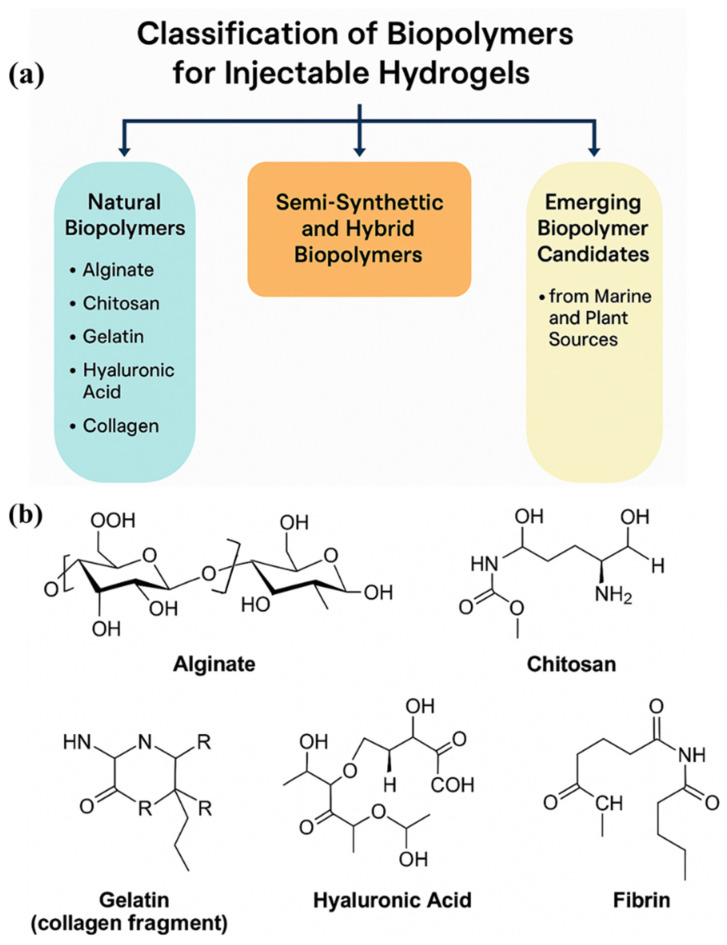
(**a**) Classification of Biopolymers Used in Injectable Hydrogel Formulations. (**b**) The molecular structures of key natural biopolymers commonly employed in the fabrication of injectable hydrogels, including alginate, chitosan, gelatin (collagen fragment), hyaluronic acid, and fibrin.

Figure 2a presents a schematic classification of biopolymers utilized in the development of injectable hydrogels, categorizing them into natural, semi-synthetic, hybrid, and emerging biopolymer classes based on their source and modification level. Natural biopolymers, such as alginate, chitosan, gelatin, hyaluronic acid, and collagen, serve as foundational materials due to their excellent biocompatibility and ECM-mimicking capabilities. Semi-synthetic and hybrid biopolymers result from chemical functionalization or the blending of natural polymers with synthetic components, offering tailored mechanical properties, enhanced stability, and controlled degradation profiles. Additionally, the inclusion of marine- and plant-derived candidates, such as carrageenan, fucoidan, pectin, and oxidized starch, reflects a growing trend toward sustainable and functional biomaterials that exhibit unique bioactivities and eco-friendliness. This classification framework supports rational hydrogel design by aligning material characteristics with specific therapeutic goals, such as in situ gelation, targeted drug release, or scaffold-assisted tissue regeneration. Figure 2b represents the molecular structures of key natural biopolymers commonly employed in the fabrication of injectable hydrogels.

### 2.3. Emerging Biopolymer Candidates from Marine and Plant Sources

As sustainability and ecological impact become increasingly relevant in material development, biopolymers from marine and plant origins are gaining traction for use in injectable hydrogel systems. These biopolymers are attractive due to their abundance, renewability, and diverse chemical functionalities, often offering unique properties not found in traditional mammalian-derived biomaterials [52]. One marine biopolymer is fucoidan, a sulfated polysaccharide extracted from brown algae, which exhibit anti-inflammatory, anticoagulant, and anti-tumor properties, making it a valuable component for injectable systems aimed at cancer therapy and vascular regeneration [53]. Fucoidan-based injectable hydrogels can self-assemble in the presence of divalent cations, like Ca^2^^+^, or be co-polymerized with gelatin or chitosan for enhanced structural stability [54]. Carrageenan, sourced from red algae, forms thermoreversible hydrogels that can encapsulate bioactive compounds and cells, especially when combined with locust bean gum or PEG. Injectable carrageenan systems are being explored for soft tissue regeneration and mucosal drug delivery [55]. Another marine polymer, ulvan, extracted from green algae, contains rare sugars, such as rhamnose and uronic acids, offering antioxidant and immunomodulatory potential [56]. Plant-derived polymers, such as pectin, cellulose derivatives, guar gum, and starch, have gained attention due to their non-toxic nature and modifiable backbones. Pectin, extracted from citrus peels and apple pomace, forms hydrogels in the presence of calcium ions and is commonly used in colon-targeted drug delivery systems [57]. Hydroxypropyl methylcellulose (HPMC) and carboxymethyl cellulose (CMC) are chemically modified cellulose variants capable of forming injectable gels upon hydration or through physical crosslinking. These materials are particularly valuable in ophthalmic and dermal applications, where minimal invasiveness and shear-thinning behavior are desirable [58]. Starch-based injectable hydrogels, though less explored, demonstrate promising shear-thinning behavior and drug-loading efficiency when modified with polyethyleneimine or poly(vinyl alcohol) [59]. Marine and plant-derived biopolymers often require functionalization or blending to overcome limitations, like poor mechanical strength or rapid degradation. Strategies include oxidation, grafting with acrylate or methacrylate groups, and co-formulation with synthetic or protein-based polymers [60]. For example, oxidized pectin can form injectable Schiff-base hydrogels with amine-rich polymers, such as gelatin or chitosan, for wound healing and periodontal regeneration [61]. Similarly, fucoidan–collagen composites have been developed for angiogenic injectable scaffolds, enhancing endothelial cell migration and blood vessel formation [62]. Unlike animal-derived polymers, marine and plant biopolymers offer lower immunogenic risk, vegan compliance, and ethical acceptability, which may streamline clinical approvals. Moreover, their abundance in agro-industrial waste adds economic and environmental value [63]. Continued research is needed to standardize the extraction, purification, and modification techniques of these biomaterials to ensure reproducibility. Additionally, multi-scale modeling and biological evaluation are essential to understand their interactions with host tissues and immune responses [64]. Emerging areas include marine-microbiome-inspired hydrogel systems, plant exosome-loaded hydrogels, and responsive materials for precision drug release, which could revolutionize the future of eco-friendly and sustainable injectable biomaterials [65].

Table 2 provides a comparative analysis of semi-synthetic, hybrid, marine, and plant-derived biopolymers, highlighting their structural origins, physicochemical features, and biomedical relevance in injectable hydrogel systems. Semi-synthetic polymers, such as gelatin methacryloyl (GelMA) and hyaluronic acid derivatives, leverage chemical modifications to enhance gelation control, cell adhesion, and mechanical tuning, making them ideal for precision tissue engineering and minimally invasive therapies. Hybrid systems, like alginate–PEG and chitosan–β-GP, integrate bioactivity with enhanced mechanical strength or thermoresponsiveness, addressing clinical needs for stability and injectability. Marine-derived polymers, including carrageenan and fucoidan, bring innate biofunctions, like anti-inflammatory and angiogenic potential, but often require mechanical reinforcement for broader applicability. Meanwhile, plant-derived materials, such as pectin, cellulose derivatives, and oxidized starch, offer eco-friendly, biodegradable options with pH sensitivity and biocompatibility, though they typically lack intrinsic cell-binding motifs. Collectively, these biopolymers exemplify the multifaceted design strategies in modern hydrogel engineering, where source origin, functionalization, and composite design converge to meet the demands of diverse biomedical applications.

## 3. Gelation Mechanisms and Injectable Formulations

Injectable hydrogels must be capable of undergoing sol–gel transitions under physiological conditions to allow for minimally invasive delivery and in situ tissue integration. The crosslinking strategy significantly impacts the mechanical integrity, degradation behavior, and cellular interaction of these hydrogels. Three major gelation approaches—physical, chemical, and enzymatic/ionic crosslinking—are commonly utilized to engineer hydrogels with tailored properties.

### 3.1. Physical Crosslinking

Physical crosslinking is a reversible, non-covalent method of hydrogel formation that relies on hydrogen bonding, hydrophobic interactions, van der Waals forces, ionic associations, and crystallization phenomena. This approach is widely adopted in biopolymer-based hydrogel systems because it generally avoids toxic chemical reagents, initiators, or catalysts, making it biocompatible and suitable for in situ biomedical applications [75]. Natural biopolymers, such as alginate, chitosan, gelatin, and carrageenan, frequently undergo physical gelation. Alginate, a marine-derived polysaccharide, gels through the formation of ionic junctions between guluronate blocks and divalent cations (e.g., Ca^2^^+^). This mechanism forms a so-called “egg-box” structure, allowing for mild gelation at body temperature without inducing inflammatory responses [76].

**Figure 3:**
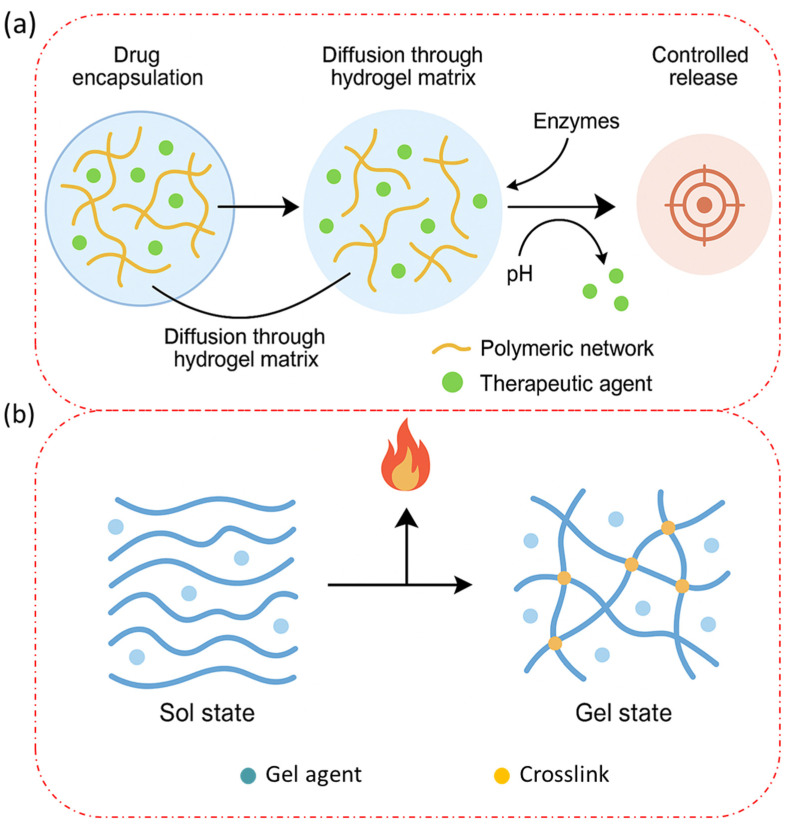
(**a**) A schematic representation of the molecular-level drug delivery mechanism of injectable biopolymer-based hydrogels. (**b**) A schematic representation of the thermo-responsive behavior of biopolymers.

Here, Figure 3a illustrates the molecular-level drug delivery mechanism of injectable biopolymer-based hydrogels, depicting key processes, such as drug encapsulation, diffusion through the hydrogel matrix, hydrogel degradation, and controlled release at the target site. The figure also includes interactions between the polymeric network and the encapsulated therapeutic agents, emphasizing stimuli-responsive behavior (e.g., pH, enzymes). Figure 3b presents a schematic representation of the thermoresponsive behavior of biopolymers (e.g., gelatin and chitosan-based systems), highlighting the sol–gel transition mechanism. The illustration captures temperature-dependent conformational changes and polymer chain interactions that enable in situ gelation after injection. Gelatin, the denatured form of collagen, forms physical gels through thermoreversible hydrogen bonding and triple helix formation. Gelatin gels below its lower critical solution temperature (~30–32 °C), a property utilized in temperature-triggered injectable systems [77]. However, physically crosslinked gelatin hydrogels tend to exhibit weak mechanical properties and rapid dissolution under physiological conditions, necessitating further stabilization. Chitosan demonstrates physical gelation via hydrophobic interactions and pH-responsive sol–gel transitions, often enhanced using neutralizing agents, like β-glycerophosphate (β-GP). These systems are particularly valuable for CNS regeneration due to their thermosensitivity and cell-compatibility [78]. The reversibility of physical gels provides advantages for controlled drug release, temporary scaffolding, and applications requiring resorption or remodeling over time. However, their mechanical fragility, poor long-term stability, and high dependency on environmental conditions (e.g., pH, temperature, ionic strength) limit their application in load-bearing tissues [79]. Recent innovations include supramolecular hydrogels formed by host–guest interactions (e.g., cyclodextrin–adamantane complexes) and self-assembling peptide-based hydrogels, which offer reversible crosslinking and bioactivity modulation for drug delivery or stem cell encapsulation [80].

### 3.2. Chemical Crosslinking

Chemical crosslinking involves the formation of covalent bonds between polymer chains, resulting in more permanent, stable, and mechanically robust hydrogel structures. This technique is ideal for fabricating injectable hydrogels with precise control over mechanical strength, degradation rate, and architecture, particularly for load-bearing applications, such as cartilage repair and intervertebral disc regeneration [81].

Several crosslinking methods are employed as follows:

- Photo-crosslinking, using photoinitiators (e.g., Irgacure 2959 or LAP) under UV or visible light exposure, is widely applied to polymers, like gelatin methacryloyl (GelMA) and methacrylated hyaluronic acid. This strategy enables spatial and temporal control over gelation, particularly in 3D bioprinting and microfluidic hydrogel systems [82].
- Click chemistry, such as thiol–ene, azide–alkyne, and Diels–Alder reactions, offers high efficiency, bioorthogonality, and minimal toxicity. Hydrogels formed via strain-promoted azide–alkyne cycloaddition (SPAAC) or Michael addition enable encapsulation of cells and drugs under mild conditions [83].
- Schiff base chemistry, often used in aldehyde-modified polysaccharides (e.g., oxidized alginate or HA) and amine-containing polymers (e.g., chitosan), facilitates rapid in situ gelation via imine bond formation, useful in wound dressing or localized therapy [84].

Despite the tunable mechanical behavior and durability, chemical crosslinking can introduce cytotoxic residue from initiators or unreacted monomers. Additionally, gelation kinetics must be optimized to avoid premature curing or incomplete gelation post-injection [85]. To address toxicity concerns, enzyme-free visible-light initiators (e.g., eosin Y) and green crosslinkers (e.g., genipin, citric acid) are gaining popularity [86]. These strategies maintain the benefits of covalent linking while reducing off-target effects and maintaining tissue compatibility.

### 3.3. Enzymatic and Ionic Gelation

Enzymatic and ionic crosslinking strategies offer bioinspired and physiologically relevant alternatives for hydrogel formation, particularly valued for their mild reaction conditions, selectivity, and minimal toxicity. These approaches are gaining traction in designing injectable systems for regenerative medicine, drug delivery, and bioinks [87]. Enzymatic crosslinking employs natural catalysts, such as horseradish peroxidase (HRP), transglutaminase, tyrosinase, and laccase, which facilitate bond formation between specific residue in the polymer chains. For instance, HRP-catalyzed crosslinking of phenol or tyramine-functionalized chitosan and gelatin produces biocompatible gels under physiological pH and temperature, suitable for cell encapsulation and drug release [88]. Tyrosinase and transglutaminase are especially relevant in protein-based hydrogels, promoting dityrosine bond formation and amide linkages, respectively. These enzymatic routes allow for the spatiotemporal regulation of gelation, supporting minimally invasive injection and tissue integration without initiating inflammatory cascades [89]. Ionic gelation involves electrostatic interactions between charged polymer chains and multivalent ions. Examples include the following:

- Alginate–Ca^2^^+^ hydrogels, with rapid gelation under divalent ion exposure and tunable porosity based on the G/M ratio.
- Chitosan–TPP (tripolyphosphate) systems, where positively charged chitosan interacts with negatively charged polyphosphates to form injectable gels for vaccine delivery or mucosal therapy [90].

These approaches are highly biocompatible, support controlled degradation, and preserve cell viability, making them ideal for stem cell therapy and injectable fillers. However, limitations include poor mechanical strength and burst degradation under physiological fluid exchange. Innovative strategies, such as dual crosslinking systems (e.g., enzymatic followed by ionic or covalent crosslinking), are being developed to balance structural integrity with biological performance [91].

### 3.4. In Situ Gelation and Thermo-Responsive Systems

In situ gelation refers to the process by which hydrogels transition from a liquid (sol) state to a gel state after being introduced into the body, typically triggered by physiological conditions, such as temperature, pH, ionic concentration, or enzymatic activity. This property is highly desirable for injectable formulations, as it allows for minimally invasive administration, accurate localization at the target site, and conformability to irregular tissue geometries, thereby enhancing therapeutic efficiency and patient comfort [92]. Among the various stimuli, temperature-responsive gelation is the most extensively studied due to its predictable behavior near physiological temperatures (37 °C). These thermo-responsive systems remain in a sol state at room temperature, facilitating syringeability, and rapidly gel upon exposure to body heat. Polymers such as poloxamers (Pluronic F127), poly(N-isopropylacrylamide) (PNIPAAm), and chitosan/β-glycerophosphate (β-GP) blends are widely used for this purpose [93]. For example, chitosan/β-GP hydrogels exhibit a critical gelation temperature close to 37 °C, offering a non-toxic and biodegradable platform suitable for drug delivery, cartilage repair, and wound healing. These hydrogels form through temperature-induced rearrangement of chitosan chains, stabilized by hydrogen bonding and hydrophobic interactions [94]. Similarly, poloxamer-based formulations undergo sol–gel transition upon warming due to micelle formation and subsequent packing, often used for ocular, nasal, and subcutaneous drug delivery [95]. Another notable thermo-responsive polymer is PNIPAAm, which exhibits a lower critical solution temperature (LCST) at ~32 °C. However, its poor biodegradability and potential cytotoxicity have led to its copolymerization with biodegradable polymers, like PEG, hyaluronic acid, or gelatin, to improve biocompatibility and mechanical properties [96]. In situ gelation can also be combined with pH-responsive behavior, as seen in alginate or gelatin methacryloyl (GelMA) systems, which form gels upon ionic interaction or light-mediated crosslinking at the site of injection. These multi-responsive formulations allow for precise spatiotemporal control over the gelation process, which is critical for cell-laden hydrogel applications in tissue engineering [97]. Moreover, enzyme-triggered in situ gelation is gaining interest due to its selectivity. For instance, the HRP/H_2_O_2_-mediated crosslinking of phenol-functionalized biopolymers enables rapid gelation in the presence of physiological peroxide levels, suitable for injectable wound sealants and drug depots [98]. The challenges of in situ forming hydrogels include ensuring sufficient mechanical strength, avoiding premature gelation, and maintaining injectability under practical conditions. Recent developments focus on tunable crosslinking kinetics, dual-responsive systems, and hybrid gelation mechanisms to improve robustness and performance [99].

Table 3 provides a comparative framework of various gelation mechanisms employed in injectable biopolymer-based hydrogels, highlighting their functional triggers, polymeric constituents, advantages, and inherent limitations. Physical and ionic crosslinking strategies are widely favored for their simplicity and biocompatibility, but often suffer from limited mechanical robustness, restricting their long-term applications in load-bearing tissues. Chemical and enzymatic crosslinking offer superior mechanical stability and spatial–temporal control, especially in tissue engineering and regenerative medicine, but require stringent conditions and can introduce cytotoxic crosslinkers or enzyme cost issues. Thermo-responsive systems are ideal for minimally invasive therapies, undergoing sol–gel transitions under physiological conditions, but their thermal instability can be a drawback for systemic applications. Self-healing and shear-thinning hydrogels, by contrast, provide mechanical adaptability and injectability, rendering them especially promising for soft tissue repair, biosensors, and dynamic physiological environments. These advanced systems underscore the necessity of multi-functional, hybrid gelation approaches to overcome the limitations of single mechanisms and to optimize clinical translational potential. Therefore, the choice of gelation method must align with the specific therapeutic objective, administration site, and degradation kinetics for optimized outcomes.

### 3.5. Self-Healing and Shear-Thinning Properties

The self-healing and shear-thinning capabilities of injectable hydrogels represent a cutting-edge frontier in biomaterials design, especially for applications requiring mechanical resilience, repeated deformation, or integration with dynamic biological environments. Self-healing hydrogels can autonomously repair structural damage or re-establish network connectivity after mechanical disruption, a feature inspired by natural tissues. This functionality arises from dynamic and reversible interactions within the hydrogel network, such as the following:

- Hydrogen bonds;
- Ionic interactions;
- Host–guest inclusion complexes;
- Dynamic covalent bonds (e.g., imine, disulfide, boronate ester bonds) [107].

In biopolymer-based systems, self-healing behavior is often imparted through Schiff base linkages (formed between aldehyde and amine groups), as seen in oxidized alginate–chitosan or HA–dopamine systems. These bonds break under mechanical strain but reform spontaneously under physiological conditions, enabling structural recovery and prolonged functional lifespan [108].

Self-healing hydrogels are particularly useful in load-bearing tissues, soft robotics, biosensors, and drug depots, where mechanical integrity is critical over time. Moreover, their injectability and adaptability make them ideal candidates for post-injection remodeling, enhancing integration with surrounding tissues [109]. Shear-thinning behavior, on the other hand, allows hydrogels to flow under applied stress (such as during injection) and rapidly recover viscosity upon cessation of shear, thus promoting smooth delivery through narrow needles without compromising mechanical integrity post-injection.

This property is typically exhibited by physically crosslinked systems, including the following:

- Chitosan/β-GP hydrogels;
- Gelatin and methylcellulose-based formulations;
- Peptide- or protein-based supramolecular hydrogels [110].

Shear-thinning is highly beneficial in 3D bioprinting, minimally invasive surgery, and cell delivery, allowing for precise patterning and structural fidelity without additional support matrices. Hybrid hydrogels integrating self-healing and shear-thinning capabilities have shown promising results in dynamic environments. For example, hydrogels composed of HA–phenylboronic acid and poly(vinyl alcohol) exhibit both features, making them ideal for cartilage repair or wound dressing, where repeated movement and healing are involved [111]. Recent studies have also demonstrated the utility of rheology tuning, where molecular weight, crosslink density, and functional group availability are optimized to balance injectability, recovery speed, and bioactivity [112]. Limitations include potential over-softening, which may compromise mechanical robustness, and slow recovery kinetics in some self-healing systems. Addressing these through dual-network strategies, multi-stimuli responsiveness, and nanocomposite reinforcement are active areas of research [113].

**Figure 4:**
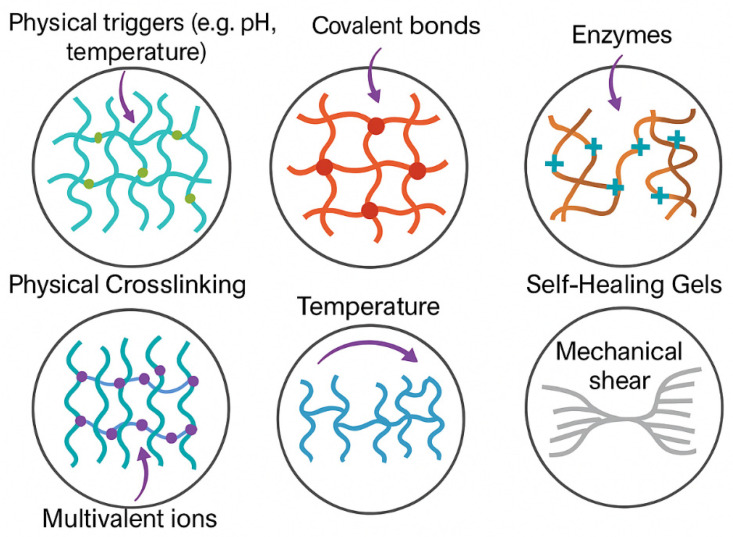
Gelation Mechanisms in Injectable Hydrogels.

The schematic illustration in Figure 4 highlights the primary gelation mechanisms leveraged in injectable hydrogel systems, emphasizing their unique physicochemical interactions and functional behaviors. Physical crosslinking involves non-covalent interactions, such as hydrogen bonding or hydrophobic effects, triggered by environmental cues, like pH or temperature changes, enabling reversible and stimuli-responsive gel formation. By contrast, chemical crosslinking relies on the formation of covalent bonds, offering enhanced mechanical stability and durability through precise crosslinker integration. Enzymatic gelation utilizes biologically relevant catalysts, like transglutaminase or horseradish peroxidase, to initiate polymer network formation under mild conditions, often improving biocompatibility. Ionic gelation occurs through electrostatic interactions between multivalent ions (e.g., Ca^2^^+^) and anionic polymers, common in alginate-based systems. Thermo-responsive gels exploit temperature shifts to transition between sol and gel phases, exemplified by PNIPAAm-modified hydrogels. Lastly, self-healing and shear-thinning gels exhibit reversible dynamic interactions, such as Schiff-base linkages or supramolecular assemblies, allowing them to flow under stress and reform their network afterward—critical for minimally invasive delivery. This figure underscores how the choice of gelation mechanism directly influences hydrogel behavior, tailoring it for specific biomedical applications, like tissue regeneration, drug delivery, and 3D bioprinting.

## 4. Design Strategies for Biomedical Applications

### 4.1. Rheological and Mechanical Property Optimization

Rheological and mechanical properties play a central role in determining the functional performance of injectable hydrogels in biomedical settings. The hydrogel must exhibit an appropriate viscoelastic profile to ensure ease of injection and structural integrity post-administration. Rheology refers to the deformation and flow behavior of the material under stress, particularly relevant for shear-thinning systems which reduce in viscosity upon application of the shear, making them easier to inject through narrow-gauge needles [114]. Shear-thinning properties facilitate minimally invasive administration and rapid recovery of the hydrogel structure once the shear is removed. Mechanical optimization, on the other hand, involves tuning the parameters, such as compressive modulus, tensile strength, and toughness, to match the native tissue environment. For instance, hydrogels intended for cartilage or bone regeneration must withstand higher mechanical loads compared to those for neural applications. Strategies such as dual crosslinking (physical and chemical), incorporation of nanofillers (e.g., graphene oxide, silica nanoparticles), and tuning polymer concentration are employed to reinforce the mechanical profile [115]. Hybrid hydrogels that combine natural and synthetic polymers can offer a balance between bioactivity and robustness. Rheological measurements, including storage modulus (G’), loss modulus (G”), and complex viscosity (η*), are key indicators to assess the mechanical readiness for in vivo performance. The interplay between rheology and mechanical strength also governs cell behavior. For example, stem cell fate can be influenced by substrate stiffness, where softer gels promote neurogenic differentiation and stiffer gels support osteogenesis [116]. Therefore, material engineers must fine-tune hydrogel mechanics to optimize both injectability and biological performance.

### 4.2. Biodegradability and Biocompatibility Considerations

Biodegradability and biocompatibility are fundamental requirements for any injectable hydrogel intended for clinical application. Biocompatibility ensures that the material does not provoke adverse immune responses or cytotoxicity, whereas biodegradability refers to the hydrogel’s ability to break down into non-toxic byproducts that are metabolized or excreted by the body over time [117]. Natural biopolymers, such as gelatin, chitosan, and hyaluronic acid, inherently possess high biocompatibility due to their structural similarity to extracellular matrix components. These materials are often enzymatically degradable, making them particularly suitable for tissue regeneration applications where matrix remodeling is critical [118]. Synthetic components, like PEG, can be engineered for controlled degradation by incorporating cleavable linkages (e.g., ester, disulfide) that respond to hydrolytic or enzymatic conditions. Controlled biodegradation is vital to match the pace of tissue healing. A hydrogel that degrades too quickly may fail to provide structural support, while a slow-degrading material might hinder natural tissue remodeling or cause chronic inflammation. Moreover, degradation kinetics influence drug release profiles and cellular infiltration, thereby affecting therapeutic outcomes [119]. To validate biocompatibility, in vitro cytotoxicity assays (e.g., MTT, Live/Dead), hemocompatibility testing, and in vivo biocompatibility studies (e.g., histological analysis, cytokine profiling) are essential. Regulatory considerations from agencies, such as the FDA, mandate stringent testing protocols before clinical translation. Therefore, designing injectable hydrogels with a fine-tuned balance of biocompatibility and degradation is imperative for their safe and effective use in biomedical therapies [120].

### 4.3. Incorporation of Bioactive Molecules

The incorporation of bioactive molecules into injectable hydrogels elevates their therapeutic efficacy by promoting cell signaling, tissue regeneration, and site-specific healing [121]. One of the most widely studied classes of bioactive molecules are growth factors like vascular endothelial growth factor (VEGF), bone morphogenetic proteins (BMPs), and transforming growth factor-beta (TGF-β), which direct angiogenesis, osteogenesis, and chondrogenesis, respectively. Controlled encapsulation and sustained release of these biomolecules enhance their biological activity while minimizing systemic toxicity [122]. Strategies such as covalent tethering, physical entrapment, and affinity-based interactions (e.g., heparin-binding) have been employed to modulate the spatial and temporal release of bioactive cues. Peptide-functionalized hydrogels (e.g., RGD, IKVAV motifs) offer biointeractive surfaces that mimic cell–ECM interactions, enhancing cell adhesion and proliferation. Nucleic acid delivery, such as siRNA or DNA, embedded in hydrogels can be used for gene silencing or expression, offering novel therapeutic avenues, particularly in cancer and regenerative medicine [123]. Importantly, the integration of bioactive agents should not compromise the mechanical and rheological properties of the hydrogel. Co-delivery systems involving combinations of bioactive molecules have also shown synergistic effects in promoting complex tissue regeneration. Thus, incorporating bioactive molecules into injectable hydrogels allows for multifunctional biomaterials that combine structural support with biological instruction [124].

### 4.4. Controlled and Sustained Drug Release Behavior

Injectable hydrogels serve as ideal drug delivery platforms due to their hydrated 3D network, which allows for encapsulation and sustained release of therapeutic agents. Controlled drug release is essential to maintain effective drug concentration at the target site, to minimize side effects, and to improve patient compliance [125]. Drug release kinetics can be tailored by manipulating hydrogel mesh size, degradation rate, and interactions between the drug and the polymer matrix. For instance, hydrogels with a dense crosslinked network exhibit slower drug diffusion, while degradable hydrogels release drugs through matrix erosion. The use of affinity-based systems (e.g., cyclodextrin-drug complexes) further fine-tunes the release profiles [126]. Multi-modal release strategies, including pH-responsive, temperature-sensitive, and enzyme-degradable systems, are being developed for precision medicine. Thermoresponsive hydrogels, like PNIPAAm, can release drugs upon temperature changes, ideal for cancer therapy. pH-sensitive gels release drugs preferentially in the acidic tumor microenvironment or infected tissues [127]. The release behavior is often modeled using kinetic models, such as Higuchi, Korsmeyer–Peppas, and zero/first-order models, to predict in vivo outcomes. Long-term drug release over weeks to months has been achieved in hydrogels loaded with chemotherapeutics, antibiotics, and analgesics, showcasing their versatility [128]. Hence, injectable hydrogels are emerging as powerful vehicles for localized, sustained, and responsive drug delivery.

Table 4 provides a comprehensive comparison of various design strategies essential for optimizing injectable hydrogels for biomedical applications. The table emphasizes that each strategy plays a distinct yet interdependent role in ensuring that the hydrogel performs effectively within the body. Rheological and mechanical property optimization is critical to ensure that the hydrogel is both injectable and able to provide the necessary mechanical support once inside the body. By adjusting properties, such as viscosity and elasticity, hydrogel formulations can be tailored for specific tissues, such as bone or cartilage, where different mechanical strengths are required. Biodegradability and biocompatibility are vital considerations in minimizing adverse effects upon administration. Biocompatible hydrogels that degrade at a controlled rate facilitate seamless integration with tissue without causing inflammation or fibrosis. By contrast, bioactive molecule incorporation allows for the targeted delivery of growth factors and drugs, enhancing therapeutic efficacy. This aspect is particularly beneficial in tissue regeneration, where growth factors and peptides can stimulate specific cell behaviors and tissue healing processes. The controlled and sustained release of drugs from hydrogels is another key factor in optimizing therapeutic outcomes. By achieving sustained release profiles, these hydrogels can reduce the frequency of administration, which is particularly advantageous for chronic disease treatments. Finally, 3D structuring and injectable scaffolds represent an innovative approach to hydrogel design, allowing for complex tissue-like environments to be mimicked. The 3D structure supports cellular organization and promotes tissue-specific regeneration, enhancing the overall effectiveness of the therapeutic application. Each design strategy comes with its own set of advantages and challenges, and the optimal strategy depends on the intended application and tissue type. By fine-tuning these strategies, researchers are advancing the development of next-generation injectable hydrogels capable of addressing a wide range of medical challenges, from wound healing to organ regeneration.

### 4.5. 3D Structuring and Injectable Scaffolds

**Figure 5:**
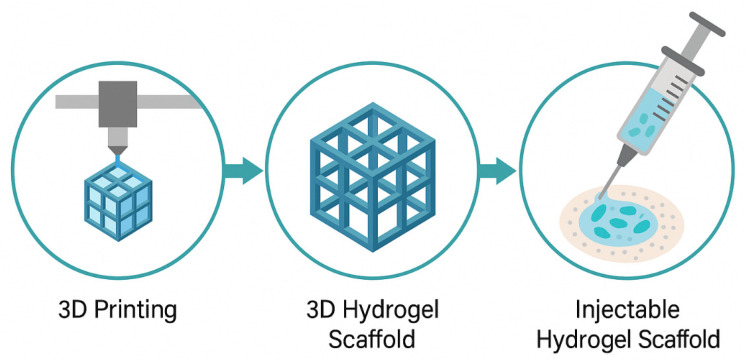
Three-Dimensional Structuring and Injectable Scaffolds.

Three-dimensional structuring of injectable hydrogels allows for the creation of biomimetic scaffolds that replicate the native architecture of tissues. This structural mimicry plays a pivotal role in directing cell behavior, facilitating nutrient diffusion, and supporting tissue regeneration [129]. Recent advances in 3D bioprinting have enabled the fabrication of complex hydrogel architectures with spatial control over cell and material placement. Bioinks composed of shear-thinning or thermoresponsive hydrogels are extruded into layered constructs that support multicellular organization. Scaffold design parameters, such as pore size, porosity, and degradation rate, are critical to ensuring vascularization and host tissue integration [130]. In situ forming scaffolds offer injectability along with mechanical support post-gelation. These systems often utilize dual-component injectables or photo-crosslinkable hydrogels activated under light exposure. Micro- and nano-patterning within the scaffold further modulates cell orientation and tissue-specific differentiation [131]. Injectable 3D scaffolds are increasingly being used for applications in cartilage repair, myocardial regeneration, and soft tissue engineering. For instance, injectable gelatin methacrylate (GelMA) scaffolds have shown promise in skin regeneration by supporting keratinocyte migration and neovascularization. The integration of stem cells or organoids into 3D injectable matrices is opening new frontiers in personalized medicine and disease modeling [132]. So, the design of 3D injectable hydrogel scaffolds bridges the gap between structural mechanics and biological functionality, enabling the development of next-generation biomaterials for regenerative and therapeutic applications. Figure 5 illustrates the process of creating injectable scaffolds with 3D structuring. It begins with 3D printing of the hydrogel to form a lattice structure. The second step shows the 3D hydrogel scaffold, which is designed for tissue engineering applications. Finally, the image transitions to the injectable form, where the scaffold is loaded with cells, making it suitable for use in regenerative medicine. This visual demonstrates the entire process, from scaffold fabrication to injection for tissue regeneration.

## 5. Biomedical Applications of Injectable Biopolymer-Based Hydrogels

Injectable biopolymer-based hydrogels have garnered immense attention as multifunctional platforms in biomedical engineering due to their high water content, tunable mechanical properties, biocompatibility, and capacity to mimic the extracellular matrix (ECM). Their injectability allows for minimally invasive administration, enabling therapeutic intervention directly at the site of disease or injury. One of the most promising domains for these hydrogels lies in targeted drug delivery, where they serve as carriers for therapeutic molecules, ensuring localized, sustained, and stimuli-responsive drug release. Their applications extend to cancer therapy, anti-inflammatory treatments, infection control, and beyond, each tailored by the hydrogel’s chemistry, crosslinking mechanism, and bioactivity.

### 5.1. Targeted Drug Delivery

Targeted drug delivery represents a paradigm shift from systemic to localized therapeutic strategies, aiming to enhance the treatment efficacy while minimizing the side effects. Injectable hydrogels, especially those based on biopolymers, offer an ideal matrix for the localized and sustained release of therapeutic agents. Their responsiveness to environmental cues, such as temperature, pH, and enzymes, makes them suitable for site-specific release, thus reducing off-target toxicity and improving drug bioavailability. Biopolymer-based hydrogels offer multiple advantages in drug delivery. Natural polymers, like alginate, chitosan, gelatin, hyaluronic acid, and their composites, can encapsulate hydrophilic and hydrophobic drugs, nucleic acids, or proteins without denaturation or degradation, ensuring therapeutic integrity [133]. Moreover, their porous structure facilitates diffusion-controlled or degradation-controlled release, making it possible to tailor the release kinetics according to the treatment needs. Hydrogels can be engineered to exhibit ”smart” behavior. For example, pH-sensitive chitosan or hyaluronic acid hydrogels can degrade in acidic tumor microenvironments or infected tissues, releasing the loaded therapeutic molecules specifically where they are needed [134]. Similarly, enzyme-sensitive hydrogels degrade in the presence of matrix metalloproteinases overexpressed in inflammatory or cancerous environments, allowing for on-demand drug release [135]. Crosslinking strategies also play a pivotal role in modulating release profiles. Physically crosslinked hydrogels (e.g., through ionic interactions or hydrogen bonding) allow for reversible and tunable drug release, whereas chemically crosslinked hydrogels provide structural robustness and sustained drug delivery over weeks to months [136]. Additionally, injectable formulations facilitate site-specific delivery by conforming to the shape of the targeted lesion post-injection. This characteristic is especially beneficial in irregularly shaped defects, such as bone voids or tumor resection cavities. Furthermore, dual-loaded systems, where hydrogels encapsulate both drugs and growth factors, have emerged for combinatorial therapy (e.g., antibiotics and tissue-regenerating agents) [137].

#### 5.1.1. Cancer Therapeutics

Cancer therapy often suffers from nonspecific systemic distribution of chemotherapeutic agents, leading to off-target toxicity and limited therapeutic efficacy. Injectable biopolymer-based hydrogels provide a promising alternative by facilitating localized intratumoral or peritumoral drug delivery. These hydrogels can encapsulate chemotherapeutic drugs, immune modulators, or gene therapies and release them directly within the tumor microenvironment (TME), thereby maximizing therapeutic effects while minimizing systemic exposure [138]. Hyaluronic acid (HA)-based hydrogels have been widely used in cancer therapy due to their natural affinity to CD44 receptors, which are overexpressed on many cancer cells. HA hydrogels, when loaded with drugs, like doxorubicin or paclitaxel, can preferentially target cancer cells via receptor-mediated uptake, thus enhancing treatment specificity [139]. Moreover, the injectability of these systems allows for repeated administration and reloading, especially beneficial in recurrent or metastatic tumors. One major challenge in cancer therapy is the heterogeneity of the tumor microenvironment, which includes hypoxia, acidic pH, and enzymatic overexpression. To tackle this, smart hydrogels have been designed to respond to TME-specific triggers. For instance, chitosan-based thermosensitive hydrogels loaded with cisplatin show in situ gelation upon injection, followed by controlled degradation in response to acidic pH and enzymatic stimuli, ensuring site-specific drug release [140]. Moreover, co-delivery systems using injectable hydrogels can encapsulate both chemotherapeutics and immunomodulatory agents. For example, gelatin-based hydrogels carrying doxorubicin and immune checkpoint inhibitors have shown synergistic antitumor effects by promoting immunogenic cell death while enhancing immune activation [141]. Similarly, liposomal drugs encapsulated within alginate or collagen-based hydrogels provide dual-layer protection and controlled release, increasing the therapeutic index [142]. Injectable hydrogels have also been employed as post-surgical tumor recurrence barriers, forming a protective depot at the surgical site loaded with anticancer agents to suppress residual tumor cell growth [143]. They have been integrated with nanoparticles, photothermal agents, or siRNA to enable multimodal therapy—combining chemotherapy, gene therapy, and hyperthermia—for resistant and aggressive tumors, like glioblastoma and pancreatic cancer [144].

In a recent preclinical study, an injectable in situ-forming hyaluronic acid–dopamine hydrogel loaded with epirubicin and Fe_3_O_4_ nanoparticles demonstrated remarkable efficacy in hepatocellular carcinoma (HCC) treatment. The hydrogel system not only allowed for pH-responsive drug release in the acidic tumor microenvironment but enabled magnetic hyperthermia, thereby combining chemotherapy and thermal ablation for synergistic tumor eradication [145]. Another noteworthy example involved a fibrin-based injectable hydrogel system co-delivering gemcitabine and TRAIL (tumor necrosis factor-related apoptosis-inducing ligand) for the treatment of pancreatic cancer. This dual-delivery platform facilitated sustained drug release and significantly enhanced apoptosis in resistant pancreatic tumor models, prolonging survival in xenograft-bearing mice [146]. Furthermore, researchers have developed a gelatin–methacrylate hydrogel embedded with gold nanorods and doxorubicin, which was injected into breast tumor sites to enable photo-responsive drug release under near-infrared (NIR) light. This approach not only improved intratumoral drug retention but enabled spatially controlled drug release and photothermal effects, effectively shrinking tumors without damaging the surrounding tissue. Collectively, these examples highlight how molecular engineering of biopolymer-based hydrogels can yield precision-targeted and multimodal cancer therapies, improving clinical translation potential.

#### 5.1.2. Anti-Inflammatory and Antimicrobial Delivery

Chronic inflammation and microbial infections pose significant challenges in regenerative medicine and postoperative care. Injectable hydrogels have emerged as effective platforms for the localized, sustained release of anti-inflammatory and antimicrobial agents. Their ability to conform to irregular wound geometries and provide a moist environment further enhances their utility in wound healing, orthopedic infections, and implant-associated inflammation [147]. Biopolymer hydrogels, such as alginate and gelatin, can be loaded with antibiotics (e.g., vancomycin, gentamicin) or anti-inflammatory agents (e.g., dexamethasone, curcumin) and injected directly into the inflamed or infected site. These hydrogels not only reduce systemic side effects but ensure high local drug concentrations, critical for eliminating biofilms and resistant bacteria [148]. In recent years, antimicrobial peptide (AMP)-loaded hydrogels have shown promise due to their broad-spectrum activity and low resistance development. Chitosan, known for its intrinsic antimicrobial properties, has been combined with AMPs and silver nanoparticles to create injectable formulations capable of tackling multidrug-resistant bacteria [149]. Similarly, plant-based hydrogels derived from guar gum or cellulose have been developed as eco-friendly and biocompatible alternatives for infection control [150]. Anti-inflammatory hydrogel systems also offer prolonged protection in diseases, such as rheumatoid arthritis and inflammatory bowel disease. For example, injectable hyaluronic acid or gelatin hydrogels carrying TNF-α inhibitors or corticosteroids provide sustained anti-inflammatory action within joint cavities or colonic tissues [151]. Enzyme-responsive hydrogels that degrade in response to inflammatory proteases further improve drug delivery precision by releasing payloads only during active inflammation phases [138]. Hydrogels also serve as dual-action agents in diabetic wound healing, delivering both antimicrobial and angiogenic agents (e.g., VEGF) to simultaneously combat infection and promote tissue regeneration [152]. Injectable formulations ensure uniform distribution across deep or tunneling wounds, improving clinical outcomes.

### 5.2. Tissue Engineering and Regenerative Medicine

#### 5.2.1. Bone and Cartilage Regeneration

Injectable biopolymer-based hydrogels have emerged as promising scaffolding materials for bone and cartilage regeneration due to their ability to fill irregular defects, support cell proliferation, and deliver osteoinductive agents locally. Bone and cartilage are complex tissues with distinct extracellular matrices (ECMs); hence, biomaterials used for their regeneration must provide mechanical stability and biological cues conducive to osteochondral repair. Natural polymers, like gelatin, chitosan, hyaluronic acid, and collagen, have shown promise due to their structural similarity to native ECM and their ability to support osteogenic differentiation [153]. In bone regeneration, injectable hydrogels are often integrated with calcium phosphate-based nanoparticles or bioactive glass to enhance osteoconductivity and mechanical strength. For example, gelatin methacryloyl (GelMA) has been functionalized with nano-hydroxyapatite (nHA) to form composite injectable gels capable of promoting osteoblast adhesion and mineralization [154]. Similarly, alginate hydrogels loaded with bone morphogenetic protein-2 (BMP-2) or platelet-derived growth factor (PDGF) have demonstrated significant improvements in bone healing in preclinical studies [155]. For cartilage regeneration, the hydrogel must provide a viscoelastic environment that mimics native cartilage. Injectable systems based on hyaluronic acid, which is a natural component of articular cartilage, have shown potential for chondrocyte encapsulation and sustained release of growth factors, like TGF-β and IGF-1 [156]. Chitosan-based thermosensitive hydrogels have also demonstrated efficacy in maintaining the phenotype of articular chondrocytes and enhancing cartilage matrix production [157]. An important consideration in osteochondral tissue engineering is the development of gradient or bilayered injectable hydrogels that can simultaneously support both bone and cartilage regeneration. These constructs typically consist of a mechanically robust lower layer for bone integration and a softer, more hydrated upper layer suitable for cartilage repair [158]. Advanced techniques, like 3D bioprinting combined with injectable bioinks, have further enabled the design of patient-specific scaffolds with zonal properties mimicking the osteochondral interface [159]. Despite these advancements, challenges persist in replicating the hierarchical architecture and vascularization of native bone. Integration with host tissue, regulation of degradation kinetics, and achieving mechanical strength sufficient for load-bearing applications remain significant hurdles. Future directions involve the incorporation of angiogenic factors, stem cells, and nanoengineered materials to enhance functional outcomes in bone and cartilage tissue regeneration [160].

#### 5.2.2. Cardiac and Neural Tissue Engineering

Cardiac and neural tissues present unique challenges due to their delicate architecture, electrical conductivity requirements, and susceptibility to inflammatory responses. Injectable hydrogels offer the dual advantage of minimally invasive delivery and adaptability to the microenvironment, making them attractive for cardiac and neural regeneration. In cardiac tissue engineering, injectable hydrogels have been employed to deliver stem cells, therapeutic proteins, or genes directly to infarcted myocardium. Biopolymers, such as alginate, collagen, and hyaluronic acid, have demonstrated the ability to preserve myocardial architecture and improve cardiac function post-myocardial infarction [161]. For instance, alginate-based hydrogels reinforced with heparin have shown promise in delivering VEGF and enhancing angiogenesis in ischemic heart tissues [162]. Injectable gelatin-based hydrogels have also been used to encapsulate mesenchymal stem cells (MSCs), which secrete paracrine factors that promote cardiac repair and reduce fibrosis [163]. Neural tissue engineering requires hydrogels that not only support cell viability and neurite extension but facilitate electrical signaling. Conductive injectable hydrogels composed of natural biopolymers integrated with conductive nanomaterials, like polypyrrole, graphene, or carbon nanotubes, have emerged as advanced platforms for neural regeneration [164]. These hydrogels enhance neural cell adhesion and synapse formation, thereby supporting functional recovery in spinal cord and brain injury models [165]. Additionally, hydrogels derived from the ECM components of neural tissue, such as laminin-functionalized hyaluronic acid or collagen, have been shown to mimic the neurogenic niche, promoting neurogenesis and neuroprotection [166]. Thermosensitive systems, such as chitosan/β-glycerophosphate gels, allow for in situ gelation in the brain cavity, enabling targeted delivery of neurotrophic factors and stem cells without surgical implantation [167]. Despite their advantages, the clinical translation of cardiac and neural injectable hydrogels require the careful consideration of immunogenicity, biodegradation, and long-term integration. Incorporating immunomodulatory agents and designing hydrogels that respond to external stimuli, such as electrical fields or pH gradients, are promising strategies under investigation [168].

#### 5.2.3. Skin and Wound Healing

Injectable hydrogels offer significant promise in the field of dermal regeneration and wound healing due to their ability to provide a moist, protective, and bioactive environment conducive to tissue repair. Skin injuries, particularly chronic wounds and burns, require materials that not only support re-epithelialization but modulate inflammation, angiogenesis, and infection. Biopolymers, such as chitosan, gelatin, and collagen, are well-known for their intrinsic wound healing properties. Chitosan, for instance, possesses antimicrobial and hemostatic properties, and has been widely used in hydrogel formulations for skin repair [169]. Collagen-based injectable gels support fibroblast migration and ECM remodeling, which are crucial for granulation tissue formation and wound contraction [170]. Gelatin hydrogels, often loaded with growth factors, like epidermal growth factor (EGF) or vascular endothelial growth factor (VEGF), can accelerate tissue granulation and neovascularization [171]. Injectable hydrogels also serve as carriers for advanced therapeutics, including stem cells, exosomes, and antimicrobial peptides. For example, injectable hyaluronic acid hydrogels encapsulating adipose-derived stem cells (ADSCs) have been shown to enhance wound closure and dermal regeneration by modulating macrophage polarization and promoting angiogenesis [172]. Similarly, antimicrobial-loaded hydrogels based on alginate or gelatin can provide the sustained release of antibiotics or silver nanoparticles, helping prevent infections in chronic wounds [173]. An emerging trend in this field is the use of smart or stimuli-responsive hydrogels that respond to local wound conditions, such as pH or enzyme activity. These hydrogels release therapeutic agents only when needed, enhancing efficacy and reducing side effects [174]. Moreover, the development of printable injectable hydrogels for skin bioprinting allows for the precise deposition of cells and bioactive molecules directly onto wounds, offering personalized regenerative therapies [175]. Challenges in clinical translation include ensuring the structural integrity of the gel in dynamic wound environments, achieving optimal degradation rates, and preventing immune rejection. Ongoing research is focused on hybrid formulations combining natural and synthetic polymers, enabling tunable properties while retaining biological functionality [176].

**Figure 6:**
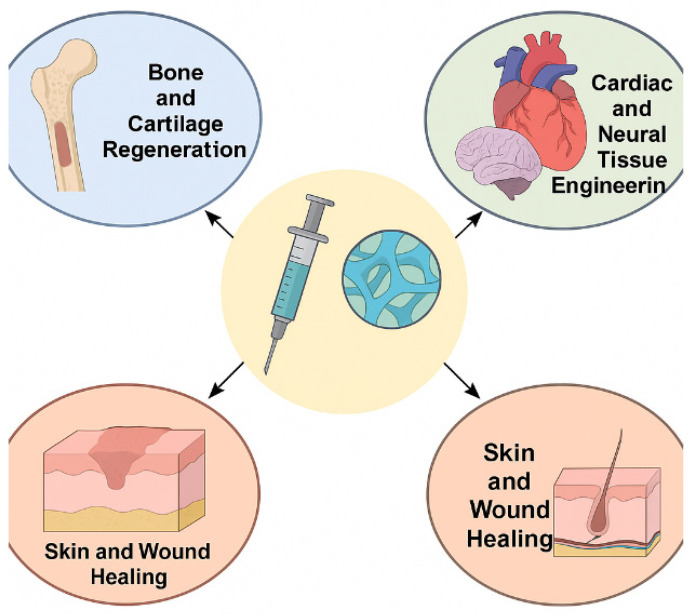
Biomedical Applications of Injectable Biopolymer-Based Hydrogels in Tissue Engineering and Regenerative Medicine.

Figure 6 illustrates the versatile roles of injectable biopolymer-based hydrogels in key areas of tissue engineering and regenerative medicine. Central to the diagram is a syringe delivering hydrogel, symbolizing the minimally invasive nature of these materials. Arrows extend toward specific applications: bone and cartilage regeneration, cardiac and neural tissue engineering, and skin and wound healing. Each domain highlights how hydrogels function as scaffolding platforms, delivering cells or bioactive molecules while providing structural support and promoting tissue repair in a controlled and localized manner.

### 5.3. Injectable Platforms for Gene and Cell Delivery

Injectable biopolymer-based hydrogels offer a highly adaptable and protective environment for delivering therapeutic genes and cells, enabling localized and sustained treatment approaches for a wide range of diseases and regenerative applications. Their ability to mimic the extracellular matrix (ECM) and provide mechanical and biochemical support makes them ideal platforms for enhancing transfection efficiency and cellular retention at target sites. In gene delivery applications, hydrogels can act as reservoirs for plasmids, small interfering RNA (siRNA), microRNA (miRNA), or viral vectors. Encapsulation of genetic materials within hydrogels ensures a controlled release profile, protection from enzymatic degradation, and reduced systemic toxicity. For instance, thermosensitive hydrogels, such as those based on chitosan or pluronic F127, have shown promising results in achieving localized gene transfer in vivo, especially in cancer therapy and vascular regeneration models [177]. These hydrogels can undergo sol-to-gel transition at physiological temperatures, enabling minimally invasive injection and subsequent solidification in situ. From a cellular delivery perspective, injectable hydrogels can encapsulate stem cells, progenitor cells, or genetically modified cells to facilitate tissue regeneration and functional restoration. Natural biopolymers, such as alginate, hyaluronic acid, and collagen, are extensively used due to their high biocompatibility and ability to support cell viability, proliferation, and differentiation. For example, mesenchymal stem cells (MSCs) encapsulated in alginate–gelatin composite hydrogels demonstrated enhanced survival and differentiation into chondrogenic lineages for cartilage regeneration [178]. In neural repair applications, injectable hyaluronic acid-based matrices have facilitated axonal growth and improved integration of transplanted neural stem cells into host tissues [179]. An important advancement in this domain is the development of bioresponsive hydrogels that react to cellular or environmental cues (e.g., pH, enzymatic activity, or oxidative stress) to release genes or support cell migration. Such smart systems are being explored to optimize tissue-specific gene expression, modulate immune responses, and enable temporally controlled differentiation of transplanted cells [180]. Additionally, hydrogels engineered with adhesion ligands and growth factors further mimic the natural ECM, thereby enhancing the interaction between encapsulated cells and the scaffold microenvironment. Despite these advancements, several challenges remain in optimizing the mechanical integrity, degradation rate, and immunological compatibility of hydrogels for clinical applications. Moreover, the standardization of delivery protocols and scaling up manufacturing under Good Manufacturing Practice (GMP) conditions are essential for translating these technologies to human trials.

A compelling example of gene delivery using injectable hydrogels is the development of chitosan–PEG-based thermoresponsive hydrogels embedded with polyethyleneimine (PEI)–plasmid DNA complexes for angiogenic gene therapy. Upon injection into ischemic limb models, these hydrogels enabled sustained plasmid release encoding for vascular endothelial growth factor (VEGF), resulting in enhanced neovascularization and improved limb perfusion [181]. In the context of cell delivery, injectable fibrin–genipin hybrid hydrogels have been utilized to deliver cardiac progenitor cells (CPCs) into infarcted myocardium. This system provided mechanical support while promoting CPC survival and integration, leading to improved cardiac function and reduced scar tissue formation in myocardial infarction models [182]. Additionally, hyaluronic acid–tyramine hydrogels incorporating CRISPR/Cas9-loaded nanoparticles have been used to facilitate localized genome editing in inflammatory bowel disease models by targeting pro-inflammatory genes, demonstrating effective gene silencing with minimal systemic side effects. Another advanced approach involves alginate–RGD peptide-modified hydrogels for the encapsulation of induced pluripotent stem cell-derived neural progenitor cells (iPSC-NPCs), which significantly enhanced cell survival, differentiation, and axonal extension in spinal cord injury models [183]. These examples emphasize the versatility of injectable biopolymer-based hydrogels as functional carriers for gene and cell therapies across a range of disease models, demonstrating both localized efficacy and translational promise.

### 5.4. Use in Minimally Invasive Surgery and Localized Therapy

Injectable biopolymer-based hydrogels have become a cornerstone for advancing minimally invasive surgery (MIS) and localized therapy, offering targeted, site-specific interventions with reduced systemic side effects and improved patient compliance. These hydrogels are specifically formulated to be administered through small-gauge needles or catheters, enabling their application in confined or delicate anatomical regions, such as the spine, brain, eye, or joints. In MIS procedures, hydrogels serve as space fillers, sealants, tissue adhesives, and hemostatic agents. For example, chitosan-based hydrogels have been employed as effective hemostats in laparoscopic procedures due to their rapid gelation and ability to promote platelet aggregation [184]. Similarly, collagen-based formulations are widely used in reconstructive surgeries to support tissue volume restoration and wound closure, minimizing the need for sutures or staples [185]. Localized therapeutic delivery using injectable hydrogels has seen significant adoption in oncology, musculoskeletal repair, and chronic inflammatory conditions. Hydrogels loaded with chemotherapeutics or biologics provide a depot for sustained drug release at the tumor site, thereby reducing systemic toxicity and improving efficacy. For instance, thermo-responsive PEG–polyester hydrogels have demonstrated the effective intratumoral delivery of paclitaxel and doxorubicin in preclinical cancer models [186]. In orthopedics, injectable calcium phosphate or gelatin-based hydrogels have been utilized for vertebroplasty and cartilage repair by promoting in situ hardening and mechanical reinforcement post-injection [187]. These systems often include osteoconductive or osteoinductive agents, such as bone morphogenetic proteins (BMPs) or hydroxyapatite nanoparticles, to enhance bone regeneration. Moreover, in cardiovascular interventions, self-healing and conductive hydrogels are being explored for repairing myocardial infarctions and vascular defects. For example, hyaluronic acid–polypyrrole composites can be injected into infarct zones to provide electrical conductivity, promote angiogenesis, and reduce infarct size [188]. An emerging focus is the development of image-guided injectable hydrogel systems that can be monitored using MRI, CT, or ultrasound. These formulations may include contrast agents or magnetic nanoparticles, facilitating real-time visualization and accurate placement during surgical procedures [189]. The ability of injectable hydrogels to conform to complex geometries, deliver therapeutic agents locally, and integrate with host tissues makes them highly suitable for a broad range of MIS applications. However, further clinical validation and the establishment of long-term safety profiles are necessary for broader adoption in surgical practice.

Table 5 presents a comparative analysis of various biomedical applications of injectable biopolymer-based hydrogels across therapeutic domains. Each application utilizes tailored hydrogel formulations—ranging from natural polymers, like hyaluronic acid (HA) and chitosan, to semi-synthetic derivatives, like GelMA and PEG composites—to meet specific clinical goals. For example, hydrogels used in cancer therapeutics focus on stimuli-responsive delivery to enhance tumor targeting, while formulations for tissue regeneration emphasize biocompatibility and mechanical support to facilitate cellular integration and matrix remodeling. In gene and cell delivery, hydrogels act as protective and sustained-release vehicles, improving transfection efficiency and stem cell engraftment, respectively. Minimally invasive surgery and localized therapies benefit from thermosensitive or ligand-modified systems that gel in situ and adapt to complex anatomical sites. The referenced studies substantiate the multifunctionality and clinical potential of each system, emphasizing the adaptability of injectable hydrogels in next-generation biomedical interventions.

## 6. Challenges and Limitations

### 6.1. Mechanical Fragility and Structural Instability

Injectable biopolymer-based hydrogels, while highly promising for biomedical applications due to their biocompatibility, tunable degradation, and bioactivity, often suffer from intrinsic mechanical weaknesses. One of the most significant challenges is their low mechanical strength and structural instability, which limit their performance in load-bearing tissues, such as bone or cartilage. Natural biopolymers, like hyaluronic acid, gelatin, and alginate, though advantageous for their biological properties, inherently lack robust mechanical integrity [199]. Hydrogels must endure physiological stresses once administered in vivo, especially in dynamic environments, such as joints or cardiac tissue. Many biopolymer-based hydrogels, upon injection, form weak gels that may degrade prematurely or fail to maintain their structural conformation under mechanical loading. For instance, physically crosslinked hydrogels, which rely on reversible interactions, such as hydrogen bonding or hydrophobic interactions, often exhibit rapid erosion or structural collapse under stress [200]. Efforts to overcome this fragility include the incorporation of reinforcing agents, such as nanoclays, carbon nanomaterials, or synthetic polymers, like PEG or PCL, into the hydrogel matrix. Hybrid systems have shown enhanced elasticity and compressive strength, particularly in cartilage regeneration studies [201]. Another approach involves dual-network hydrogels, where an initial loosely crosslinked polymer provides flexibility, and a second tightly crosslinked network offers strength [202]. However, reinforcing hydrogels must be carefully balanced with maintaining injectability and biocompatibility. Over-crosslinking or the inclusion of rigid nanofillers can compromise cell viability or prevent minimally invasive delivery. In addition, material fatigue under cyclic mechanical loading remains a major obstacle for long-term stability. This becomes especially relevant for tissue scaffolds intended to remain functional over weeks or months [203]. Thus, while promising strategies exist to reinforce these hydrogels, achieving a balance between mechanical robustness, biodegradability, and biological function is still an unresolved challenge, especially for applications involving dynamic mechanical environments.

### 6.2. Sterilization and Storage Constraints

Sterilization is an essential prerequisite for any biomedical product, yet biopolymer-based injectable hydrogels pose significant challenges in this regard. Conventional sterilization methods, such as autoclaving, gamma irradiation, and ethylene oxide exposure, often lead to the degradation of the polymer backbone or disruption of the gelation properties [204]. For example, alginate and gelatin can undergo hydrolysis or loss of functional groups when exposed to high heat or radiation, adversely affecting their crosslinking capabilities and mechanical properties [205]. Moreover, injectable hydrogels typically contain sensitive bioactive molecules, such as growth factors, peptides, or live cells, which are highly susceptible to inactivation during harsh sterilization procedures. This complicates the scalability of formulations and restricts shelf-life. Even filtration-based sterilization is not universally applicable, especially for viscous formulations or gels containing nanoparticles or encapsulated drugs [206]. Storage presents another limitation. Many hydrogel formulations require low-temperature conditions (e.g., refrigeration or freezing) to prevent premature gelation or degradation. Thermoresponsive systems, for instance, may begin crosslinking at room temperature, leading to gelation inside syringes prior to administration. Moreover, freeze–thaw cycles can lead to phase separation or structural damage in formulations containing liposomes or emulsions [207]. To address these constraints, strategies such as lyophilization followed by rehydration, use of stabilizing agents (e.g., trehalose or mannitol), or in situ crosslinking post-injection are being explored. Injectable hydrogel precursors stored in separate components (i.e., two-syringe systems) and mixed at the site of administration can prolong shelf-life and prevent pre-gelation [208]. Despite these innovations, regulatory requirements demand standardized, robust sterilization protocols and long-term stability data, which remain lacking for most formulations. These issues underscore the need for interdisciplinary solutions involving materials science, pharmaceutical engineering, and regulatory compliance to fully translate hydrogel systems into clinical products [209].

### 6.3. Batch-to-Batch Variation in Biopolymers

Biopolymers derived from natural sources, such as chitosan, alginate, collagen, and hyaluronic acid, often exhibit significant batch-to-batch variability in physicochemical characteristics. This inconsistency arises due to variations in the source material (e.g., animal species, tissue type, harvesting method), extraction processes, and purification techniques [210]. Differences in molecular weight, degree of deacetylation (for chitosan), or guluronic/mannuronic acid content (for alginate) can drastically influence the gelation behavior, mechanical strength, degradation kinetics, and biological interactions of the resulting hydrogel [211]. Such variability poses challenges not only for reproducible research outcomes but for regulatory approval of biopolymer-based hydrogel products. Lack of standardization leads to inconsistent crosslinking efficiency, mechanical properties, and cellular responses. For instance, variation in collagen fiber diameter or degree of fibrillogenesis can alter the hydrogel’s ability to support tissue regeneration or cellular adhesion [212]. Efforts to mitigate variability include rigorous quality control during biopolymer processing, use of chemically modified derivatives (e.g., methacrylated gelatin or oxidized alginate), or blending with synthetic polymers to improve consistency [213]. Recombinant biopolymers produced in microbial systems offer another promising route to minimize heterogeneity and contamination risks, especially for biomedical applications requiring clinical-grade reproducibility [214]. Nonetheless, complete elimination of batch-to-batch variability remains elusive, particularly when using materials derived from animal tissues. This limitation underscores the importance of establishing well-defined characterization protocols and adopting Good Manufacturing Practices (GMP) for clinical-grade biopolymer production [215].

### 6.4. Immunogenicity and Regulatory Barriers

Despite being generally considered biocompatible, many natural biopolymers are not entirely free from immunogenic responses. Animal-derived materials, such as collagen (from bovine or porcine sources) or gelatin, may elicit allergic or inflammatory reactions in susceptible individuals due to residual proteins, endotoxins, or incomplete purification [216]. Furthermore, the incorporation of bioactive agents or chemical crosslinkers (e.g., glutaraldehyde or carbodiimide) may further increase immunogenicity, cytotoxicity, or long-term foreign body reactions [217]. Another concern involves the degradation products of injectable hydrogels. For example, alginate degrades into uronic acids, which in high concentrations can cause local inflammation or osmotic imbalance. Similarly, chitosan degradation products can induce macrophage activation depending on the degree of deacetylation and molecular weight [218]. From a regulatory standpoint, injectable hydrogels fall under the complex category of combination products—requiring the stringent evaluation of safety, efficacy, sterility, biocompatibility, and long-term degradation profiles. The United States Food and Drug Administration (FDA) and European Medicines Agency (EMA) have established guidelines for hydrogel-based medical devices, but approval timelines remain lengthy and costly due to the multidisciplinary nature of the product [219]. To meet regulatory requirements, hydrogel systems must demonstrate not only batch consistency and safety in animal models, but human clinical trials with long-term follow-up. Additionally, scalability and compliance with GMP standards are mandatory for commercial translation. Many promising hydrogel formulations fail to reach the market due to challenges in aligning innovative design with strict regulatory expectations [220]. As a result, ongoing research must prioritize not only the biological performance of hydrogels but translational considerations, including immunogenicity mitigation, regulatory documentation, and industrial scalability. Future progress will likely depend on collaborative efforts between researchers, clinicians, regulatory agencies, and manufacturers to create safe, standardized, and effective hydrogel platforms for clinical use.

## 7. Future Perspectives and Emerging Trends

### 7.1. Bioinspired and Smart Responsive Hydrogels

Bioinspired hydrogels, mimicking natural extracellular matrices and biological signaling environments, are gaining momentum in next-generation therapeutic applications. These materials are engineered to respond to biological cues or environmental stimuli, such as pH, temperature, enzymatic activity, and redox conditions. The goal is to develop systems that can perform site-specific action with on-demand therapeutic release, closely emulating physiological dynamics. Smart hydrogels can undergo reversible sol–gel transitions or modulate their properties in response to stimuli, making them highly suitable for minimally invasive injectables. For instance, hydrogels incorporating temperature-sensitive polymers, such as poly(N-isopropylacrylamide) (PNIPAAm), allow for gelation upon reaching body temperature, ensuring efficient localization post-injection [221]. Similarly, pH-responsive gels can release drugs specifically in the acidic microenvironment of tumors or infected tissues, improving therapeutic efficacy and reducing off-target effects [222]. Enzyme-responsive systems have also demonstrated potential in targeting diseased tissues where overexpressed enzymes (e.g., MMPs in cancer or inflammation) degrade specific crosslinkers to trigger gel dissolution or drug release [223]. Advances in dynamic covalent chemistry have enabled hydrogels with self-healing and reversible bonding, expanding their utility for repetitive delivery or in high-stress biological environments [224]. Biomimetic systems inspired by mussel adhesive proteins, spider silk, or decellularized matrices offer improved bioactivity, adhesiveness, and structural strength [225]. These designs often integrate peptide motifs (e.g., RGD sequences) to promote cell attachment and proliferation, supporting regenerative processes. Looking ahead, the integration of synthetic biology and protein engineering with hydrogel technology may allow for programmable and adaptive materials tailored for specific tissue responses.

### 7.2. Integration with Nanotechnology and 3D Bioprinting

The convergence of nanotechnology with hydrogel engineering has led to innovative materials with enhanced mechanical strength, bioactivity, and targeted delivery capabilities. Nanomaterials, such as graphene oxide, carbon nanotubes, mesoporous silica nanoparticles, and metallic nanostructures (e.g., gold or silver NPs), have been successfully embedded in hydrogels to impart conductivity, antibacterial properties, or magnetic responsiveness [226]. This nanocomposite design allows for spatial and temporal control of therapeutic delivery, particularly for gene delivery, neuroregeneration, and cancer therapy. For instance, magnetic nanoparticles enable the remote modulation of hydrogel structure or drug release via external magnetic fields, providing non-invasive therapeutic control [227]. Parallelly, 3D bioprinting has become a transformative tool for fabricating injectable scaffolds with high spatial fidelity and patient-specific architectures. Bioprintable hydrogels (bioinks) are engineered for optimized viscosity, shear-thinning behavior, and crosslinking kinetics, allowing for extrusion-based printing into anatomical defect sites [228]. The marriage of nanomaterials and 3D bioprinting enables the design of complex hybrid structures with gradient properties, mimicking tissue heterogeneity. For instance, printing stem cells within growth factor-laden nanohybrid gels allows for localized differentiation and tissue morphogenesis [229]. Future work may focus on dynamic 4D bioprinting systems, where hydrogels evolve post-injection to adapt to the biological milieu, opening frontiers in tissue replacement and organ repair [230].

### 7.3. Personalized Medicine and Patient-Specific Gels

Personalized medicine is rapidly reshaping modern healthcare, with injectable hydrogels poised to play a central role in delivering individualized therapies. The adaptability of biopolymer hydrogels in terms of their composition, degradation rate, and release profile makes them ideal for patient-specific treatment regimens, particularly in oncology, autoimmune disorders, and wound care [231]. Emerging techniques, like patient-derived cells or organoids embedded in hydrogels, enable disease modeling and drug screening, informing real-time decisions for therapy selection [232]. Moreover, 3D printed or in situ-formed hydrogels that conform to patient anatomy—guided by MRI or CT imaging—allow for the precise repair of tissue defects or targeted drug reservoirs. Personalized hydrogels also provide a platform for gene editing tools (e.g., CRISPR-Cas9), cell therapies (e.g., CAR-T), and autologous tissue grafting, enhancing therapeutic precision and minimizing immune responses [181]. The development of “digital twin” models combining patient genomic data with hydrogel-based microphysiological systems may soon become a clinical reality, enabling predictive treatment outcomes [233]. Nonetheless, scalable production and cost-effectiveness remain key barriers. Incorporating AI-driven formulation optimization and machine learning-guided material selection could accelerate the development of next-gen hydrogels for personalized clinical applications [234].

### 7.4. Clinical Translation and Commercialization Potential

Despite promising preclinical outcomes, the clinical translation of injectable biopolymer-based hydrogels faces multifaceted challenges. Key hurdles include regulatory compliance, scale-up manufacturing, long-term safety data, and reproducibility. However, successful commercial hydrogel products, such as *Seprafilm^®^*, *Regranex^®^*, and *Cymetra^®^*, demonstrate the feasibility and market potential of these biomaterials [235]. Clinical trials involving injectable hydrogels for tissue repair, drug delivery, and even cell encapsulation (e.g., pancreatic islets for diabetes) are expanding, driven by advances in formulation, sterilization, and delivery systems. Regulatory frameworks from the FDA and EMA are evolving to accommodate combination products (e.g., drug-hydrogel or cell-hydrogel systems), though standardization of biopolymer sourcing and batch consistency remains critical [236]. Commercialization also hinges on the ability to scale up without compromising biocompatibility or performance. Emerging strategies include microfluidic synthesis for uniform gel particles, lyophilized gel kits for on-demand rehydration, and injectable pens or cartridges for patient-friendly administration [237]. Collaborations between academia, biotech firms, and pharmaceutical giants are crucial for accelerating technology transfer and reducing time-to-market. Moreover, intellectual property landscape and cost–benefit analysis must be considered, especially for personalized hydrogel formulations.

Looking forward, the success of hydrogel platforms in clinics will depend on rigorous validation, post-market surveillance, and patient-centric design. Smart packaging, digital monitoring, and feedback-enabled hydrogels may redefine their role in real-time personalized medicine [238].

## 8. Conclusions

### 8.1. Summary of Key Findings

Injectable biopolymer-based hydrogels have emerged as a transformative class of biomaterials with multifaceted applications in modern biomedicine. This review has systematically highlighted the classification of biopolymers—spanning natural, semi-synthetic, hybrid, and emerging sources—underscoring their physicochemical properties, biological compatibility, and structural versatility. The mechanisms of gelation, including physical, chemical, enzymatic, and thermo-responsive strategies, have been explored with an emphasis on their injectability, responsiveness, and clinical feasibility. Design optimization strategies were critically discussed, addressing essential criteria, such as rheology, mechanical stability, degradation profiles, bioactivity, and controlled release. These attributes are paramount for successful application in drug delivery, regenerative medicine, tissue engineering, gene and cell therapies, and localized surgical interventions. Furthermore, the integration of nanotechnology, smart materials, and 3D bioprinting has opened avenues for patient-specific treatments and personalized medicine. Biomedical applications were explored in depth, revealing the role of hydrogels in the site-specific delivery of therapeutics, tissue repair, and minimally invasive procedures. Additionally, the challenges and limitations associated with manufacturing, immune responses, regulatory pathways, and reproducibility were critically examined to identify the gaps hindering clinical translation.

### 8.2. Final Remarks on the Promise of Injectable Biopolymer Hydrogels

Injectable hydrogels based on biopolymers stand at the crossroads of innovation and clinical transformation. Their tunable architecture, biocompatibility, and capacity for functional customization make them ideal candidates for next-generation therapeutic platforms. As research advances, bioinspired designs and multifunctional composites will redefine their role from passive carriers to active participants in healing and regeneration. Despite current challenges, continuous progress in material science, bioengineering, and translational medicine is poised to address the existing barriers. The synergistic integration of stimuli-responsive behavior, patient-specific tailoring, and emerging fabrication technologies will likely push the boundaries of biomedical applications. In conclusion, injectable biopolymer hydrogels represent a promising frontier in minimally invasive therapeutics. With continued interdisciplinary collaboration, rigorous validation, and translational efforts, these smart biomaterials are expected to significantly improve patient outcomes and usher in a new era of precision medicine.

## Figures and Tables

**Table 1:** Comparison of Natural Biopolymers Used in Injectable Hydrogels.

Biopolymer	Source	Key Properties	Biomedical Applications	Limitations
**Alginate**	Brown seaweed	Ionic crosslinking, biocompatible, fast gelation	Wound healing, drug delivery, cartilage regeneration	Poor cell adhesion, mechanical weakness
**Chitosan**	Crustacean shells	Antibacterial, mucoadhesive, biodegradable	Skin repair, nerve regeneration, hemostasis	Insoluble at physiological pH
**Gelatin**	Denatured collagen	Thermoresponsive, promotes cell adhesion and migration	Tissue scaffolding, drug release, cell encapsulation	Weak mechanical properties, batch variability
**Hyaluronic Acid**	Connective tissue	ECM mimic, promotes angiogenesis, hydrophilic	Wound healing, osteoarthritis treatment, cosmetic fillers	Rapid degradation, low mechanical strength
**Collagen**	Animal tissue	Native ECM protein, supports cell growth and differentiation	Bone regeneration, skin tissue engineering	Immunogenicity (depending on source), gelation control required
**Fibrin**	Blood plasma protein	Fast gelation, supports angiogenesis and hemostasis	Wound sealants, cardiovascular applications	Rapid degradation, weak long-term mechanical support
**Silk Fibroin**	Silkworm cocoons	High mechanical strength, tunable degradation	Bone tissue, nerve repair, drug carriers	Slow gelation, requires pre-processing

**Table 2:** Comparative Analysis of Semi-Synthetic, Hybrid, Marine, and Plant-Derived Biopolymers for Injectable Hydrogel Applications.

Biopolymer Type	Source	Key Functional Properties	Common Applications	Advantages	Limitations	References
**Gelatin Methacryloyl (GelMA)**	Animal-derived (modified)	Photopolymerizable; cell adhesion sites; enzymatic degradation	Tissue engineering, wound healing	ECM mimicry; tunable stiffness; photo-crosslinkable	UV exposure risks; needs photoinitiators	[66]
**HA-derivatives (e.g., HA-CHO)**	Animal-derived (modified)	Injectable via Schiff base reactions; supports cell migration	Cartilage repair, drug delivery	Biocompatible; modifiable for in situ gelling	Rapid degradation; variable gelation	[67]
**Alginate–PEG Hybrid**	Marine + synthetic blend	Ionic/covalent crosslinking; improved mechanical strength	Bone tissue scaffolds, 3D bioprinting	Customizable rheology; dual crosslinking	Bioinert unless modified	[68]
**Chitosan–β-GP Hybrid**	Marine-derived hybrid	Thermo-responsive (sol–gel transition at ~37 °C)	Injectable scaffolds, CNS regeneration	Easy injection; physiological gelation	Low long-term mechanical strength	[69]
**Carrageenan**	Red algae (marine)	Thermoreversible gelation; sulfate groups for bioactivity	Mucosal drug delivery, wound dressing	Natural gelling; anti-inflammatory	Weak mechanical properties; low cell adhesion	[70]
**Fucoidan**	Brown algae (marine)	Anticoagulant, anti-inflammatory; ionic crosslinking	Cancer therapy, angiogenesis scaffolds	Bioactive; supports vascularization	Extraction variability; limited mechanical resilience	[71]
**Pectin**	Citrus/apple waste (plant)	Ionically crosslinkable with Ca^2^^+^; pH-sensitive	Colon-specific delivery, wound dressing	Biodegradable; widely available	Poor mechanical strength; fast degradation	[72]
**CMC/HPMC**	Cellulose derivative (plant)	Viscosity control; injectable as shear-thinning fluid	Ophthalmics, dermal fillers	Renewable; modifiable functional groups	Limited bioactivity; often inert	[73]
**Oxidized Starch Derivatives**	Corn/potato starch (plant)	Injectable; forms gels with polymers like PVA	Drug delivery, tissue adhesives	Eco-friendly; abundant source	Needs chemical functionalization for gelling	[74]

**Table 3:** Comparative Analysis of Gelation Mechanisms in Injectable Biopolymer-Based Hydrogels.

Gelation Mechanism	Trigger	Key Biopolymers	Advantages	Limitations	References
**Physical Crosslinking**	Temperature, pH, ionic strength	Gelatin, chitosan, alginate	Mild conditions, reversible, injectable, biocompatible	Poor mechanical strength, uncontrollable degradation	[100]
**Chemical Crosslinking**	Covalent bonding (photo, redox, click)	GelMA, HA-DA, PEG-aldehyde systems	Strong and stable gels, tunable mechanical properties	Toxic initiators, complex synthesis	[101]
**Enzymatic Gelation**	HRP/H_2_O_2_, tyrosinase, transglutaminase	Gelatin–phenol, HA–tyramine, fibrin	Biocompatible, site-specific, in situ gelation	Costly enzymes, sensitivity to enzymatic activity	[102]
**Ionic Gelation**	Multivalent ions (e.g., Ca^2^^+^, Fe^3^^+^)	Alginate, chitosan, carrageenan	Fast gelation, low toxicity, good for cell encapsulation	Weak mechanical integrity, ion leaching	[103]
**Thermo-Responsive Systems**	Body temperature (~37 °C)	Chitosan/β–GP, Pluronic F127, PNIPAAm hybrids	Injectable, sol-to-gel transition in vivo	Poor mechanical strength, unstable at elevated temperatures	[104]
**Self-Healing Gels**	Dynamic bonds (Schiff base, host–guest)	Oxidized alginate, HA–borate, gelatin–PEG hydrazone	Durable, injectable, regenerable network	Slow recovery in some cases, trade-off with strength	[105]
**Shear-Thinning Gels**	Mechanical shear	Chitosan blends, collagen, peptide hydrogels	Easily injectable, shape-conforming, ideal for bioprinting	Rapid breakdown under shear, recovery kinetics variability	[106]

**Table 4:** Comparative Analysis of Design Strategies for Injectable Hydrogels in Biomedical Applications.

Design Strategy	Description	Advantages	Challenges	Applications
**Rheological and Mechanical Property Optimization**	Optimizing viscosity, elasticity, and mechanical strength for ease of injection and functional support in tissue.	Improved injectability, tailored mechanical properties for specific tissues, enhanced post-injection integrity.	Balancing viscosity for injectability vs. mechanical strength, maintaining shear-thinning properties without compromising stability.	Cartilage repair, bone regeneration, neural scaffolds, tissue engineering.
**Biodegradability and Biocompatibility**	Ensuring the hydrogel degrades at a rate matching tissue healing and does not provoke an immune response.	Biocompatibility with minimal adverse reactions, controlled degradation for tissue integration.	Achieving controlled degradation without impeding healing; potential for long-term systemic effects if not properly designed.	Soft tissue regeneration, wound healing, in vivo drug delivery.
**Incorporation of Bioactive Molecules**	Loading the hydrogel with growth factors, peptides, or drugs to accelerate healing and promote cell behavior.	Enhanced tissue regeneration, targeted therapeutic effects, localized delivery of active compounds.	Maintaining bioactivity during encapsulation and release, potential for rapid clearance of bioactive agents.	Cancer therapy, bone and cartilage regeneration, stem cell therapies, gene therapy.
**Controlled and Sustained Drug Release**	Developing hydrogels that release drugs over extended periods with controlled kinetics.	Prolonged therapeutic effects, reduction in drug dosing frequency, reduced side effects.	Ensuring consistent release profile, preventing burst release, overcoming biological barriers to effective drug release.	Cancer therapy, chronic disease treatment, antimicrobial treatments, pain management.
**3D Structuring and Injectable Scaffolds**	Creating 3D structures within hydrogels to mimic natural tissues and promote cell organization, migration, and regeneration.	Mimics natural tissue architecture, supports cell growth and vascularization, adaptable to irregular defects.	Complexity of 3D patterning, maintaining cell viability, optimizing scaffold degradation for long-term healing.	Cartilage repair, wound healing, skin regeneration, organ tissue engineering, personalized medicine.

**Table 5:** Comparative Overview of Biomedical Applications of Injectable Biopolymer-Based Hydrogels.

Application Area	Hydrogel Type/Composition	Therapeutic Target	Delivery Mechanism	Advantages	Representative References
**Cancer Therapeutics**	HA-based, PEGylated chitosan, alginate–gelatin composites	Tumor-specific drug delivery (e.g., DOX, PTX)	Localized, pH/temperature-responsive in situ gelation	Reduced systemic toxicity, improved bioavailability	[190]
**Anti-inflammatory and Antimicrobial**	Chitosan–silver nanocomposites, alginate–AMP hydrogels	Inflammation, bacterial infections (e.g., wound beds)	Controlled release of anti-inflammatory or antimicrobial agents	Dual functionality: tissue regeneration and microbial suppression	[191]
**Bone and Cartilage Regeneration**	Collagen–hydroxyapatite, GelMA, alginate–BMP composites	Bone defects, cartilage repair	Injectable scaffold with osteoinductive molecules/cells	Supports osteogenesis/chondrogenesis, promotes matrix mineralization	[192]
**Cardiac Tissue Engineering**	HA–collagen, alginate–fibrin hybrid hydrogels	Myocardial infarction repair	Cell-laden or drug-loaded injectables post-MI	Enhances vascularization, promotes cardiomyocyte survival	[193]
**Neural Tissue Engineering**	Chitosan–GelMA, PEG–HA hybrid systems	Neural regeneration post-stroke or trauma	Injectable neuro-supportive matrix with growth factors	Reduces glial scarring, supports axonal growth and neural integration	[194]
**Skin and Wound Healing**	Gelatin/HA, chitosan–aloe vera, collagen–silver NP hydrogels	Chronic wounds, burns, diabetic ulcers	In situ gel with anti-microbial, anti-inflammatory agents	Promotes granulation, moisture retention, antimicrobial effects	[195]
**Gene Delivery**	PEI-functionalized gelatin, chitosan-DNA, HA-based nanoparticles	siRNA, plasmid DNA, miRNA delivery	Encapsulation or complexation within hydrogels	Protects genetic cargo, localized and sustained transfection	[196]
**Cell Delivery (Stem/Progenitor)**	GelMA, collagen–PEGDA, alginate–MSC encapsulation	Stem cell therapy for regenerative medicine	Injectable matrices for cell viability and localization	Enhances cell retention, survival, and paracrine signaling	[197]
**Minimally Invasive Surgery**	Thermosensitive chitosan, Pluronic-based composites	Postoperative drug release, tissue repair	In situ gelation in body temperature-responsive manner	Easy administration, adaptable to irregular defect geometries	[143]
**Localized Therapy**	Alginate–GelMA with targeted ligands	Site-specific chemotherapy or anti-inflammatories	Ligand-guided hydrogel deposition	Improves localization, lowers off-target effects	[198]

## Data Availability

No new data were created or analyzed in this study.
